# Suppression of glioblastoma by a drug cocktail reprogramming tumor cells into neuronal like cells

**DOI:** 10.1038/s41598-019-39852-5

**Published:** 2019-03-05

**Authors:** Longfei Gao, Shichao Huang, Hong Zhang, Wei Hua, Shunmei Xin, Lin Cheng, Wuqiang Guan, Yongchun Yu, Ying Mao, Gang Pei

**Affiliations:** 1 State Key Laboratory of Cell Biology, CAS Center for Excellence in Molecular Cell Science, Shanghai Institute of Biochemistry and Cell Biology, Chinese Academy of Sciences, University of Chinese Academy of Sciences, 320 Yueyang Road, Shanghai, 200031 China; 2 Shanghai Key Laboratory of Signaling and Disease Research, Collaborative Innovation Center for Brain Science, School of Life Sciences and Technology, Tongji University, Shanghai, 200092 China; 3 School of Life Science and Technology, ShanghaiTech University, 100 Haike Road, Shanghai, 201210 China; 4 Department of Neurosurgery, Huashan Hospital, Fudan University, 12 Middle Wulumuqi Road, Shanghai, 200040 China; 5 State Key Laboratory of Medical Genomics, Shanghai Institute of Hematology, Rui Jin Hospital, Shanghai Jiao Tong University School of Medicine, Shanghai, 200025 China; 6 Institute of Neurobiology, Institutes of Brain Science, State Key Laboratory of Medical Neurobiology and Collaborative Innovation Center for Brain Science, Fudan University, Shanghai, 200032 China; 7 State Key Laboratory of Medical Neurobiology, School of Basic Medical Sciences, Institutes of Brain Science and Collaborative Innovation Center for Brain Science, Fudan University, 131 Dong’an Road, Shanghai, 200032 China; 8 Institute for Stem Cell and Regeneration, Chinese Academy of Sciences, Beijing, 100101 China

## Abstract

Glioblastoma (GBM) is the most common and aggressive malignant tumor in adult brain. Even with the current standard therapy including surgical resection followed by postoperative radiotherapy and chemotherapy with temozolomide (Temo), GBM patients still have a poor median survival. Reprogramming of tumor cells into non-malignant cells might be a promising therapeutic strategy for malignant tumors, including GBM. Based on previous studies using small molecules to reprogram astrocytes into neuronal cells, here we further identified a FTT cocktail of three commonly used drugs (Fasudil, Tranilast, and Temo) to reprogram patient-derived GBM cells, either cultured in serum containing or serum-free medium, into neuronal like cells. FTT-treated GBM cells displayed a neuronal like morphology, expressed neuronal genes, exhibited neuronal electrophysiological properties, and showed attenuated malignancy. More importantly, FTT cocktail more significantly suppressed tumor growth and prolonged survival in GBM patient derived xenograft than Temo alone. Our study provided preclinical evidence that the neuronal reprogramming drug cocktail might be a promising strategy to improve the existing treatment for GBM.

## Introduction

Glioblastoma (GBM) is the most prevalent and aggressive malignant tumor in adult brain and one of the most challenging malignancies in the oncology. For many years, surgical resection and postoperative radiotherapy had been the standard treatment for GBM, which resulted in a poor median survival of about 12 months^1,2^. Currently, the addition of temozolomide (Temo) to surgery and radiotherapy has become the standard first-line treatment for GBM, but with an increase of the median survival for only about 2.5 months^1,2^. Despite the number of FDA-approved drugs for cancer treatment has increased substantially over the past decades and much progress has been made in the molecular and cellular profiling of GBM, there are still limited effective therapies against GBM.

As a cutting-edge technology, transcription factor (TF)-mediated cell reprogramming holds great promise for cell therapy and regenerative medicine. For example, neuronal TFs reprogrammed astrocytes into neuronal cells^3,4^, offering a new avenue to regenerate neuronal cells and reverse deleterious astrocytes. Moreover, tumorigenicity of B cell leukemia or GBM was impaired with TFs reprogramming tumor cells into macrophages or neuronal like cells^5–10^, suggesting that using this technology to reprogram tumor cells into non-malignant cells might provide a potential therapeutic strategy for malignant tumors. With unique advantages in safety considerations and biological effects, small molecules are ideal alternatives for TFs to induce cell reprogramming. Previous studies have demonstrated that small molecules successfully induced cell reprogramming without the introduction of ectopic genes^11–17^. Among these studies, we found that mouse and human astrocytes were reprogrammed into neuronal cells with specific small molecules^11,13^. In this study, we further identified a cocktail of three commonly used drugs to reprogram patient-derived GBM cells into neuronal like cells. Compared with Temo alone, this cocktail also exerted a more potent effect in suppression of tumor growth and promotion of survival in GBM patient derived xenograft (PDX). Thus, the drug cocktail identified in a reprogramming logic might improve the existing treatment against GBM.

## Results

### Identification of neuronal reprogramming drug cocktail

**Figure 1:**
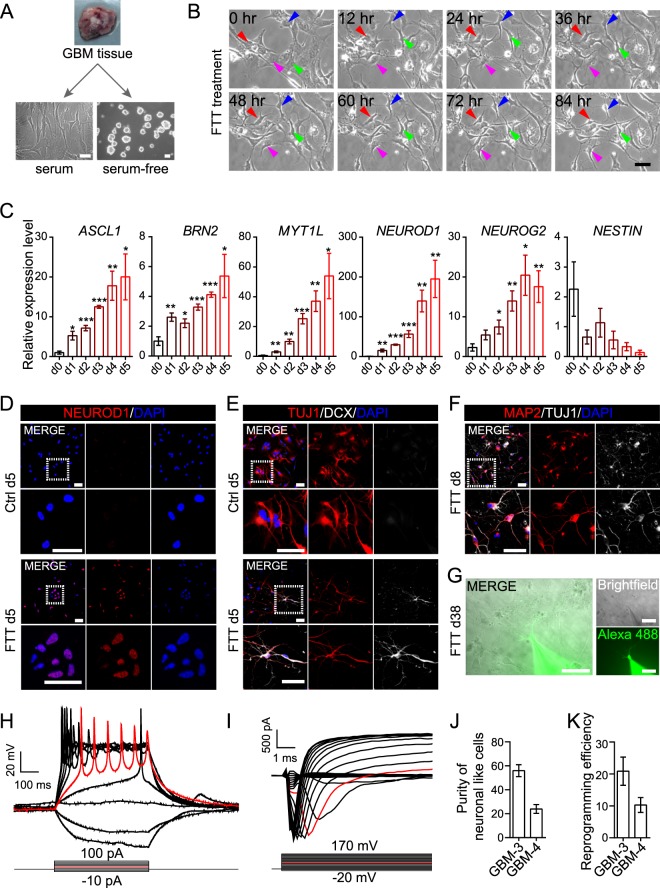
A drug cocktail (FTT) reprogrammed serum cultured GBM cells into neuronal like cells. (**A**) Schematic diagram showing that GBM cells were cultured as adherent monolayer in serum-containing medium or as sphere in serum-free medium. (**B**) Time lapse images showing GBM cell morphology at indicated timepoint under FTT treatment. Arrowheads mark example cells with morphology change along the induction process. Arrowheads with the same color indicated the same cell at different timepoint. (**C**) Analysis of the expression of *ASCL1*, *BRN2*, *MYT1L*, *NEUROD1*, *NEUROG2*, and *NESTIN* on FTT-treated GBM cells. *P* values versus d0 were calculated with two-tailed student’s t test. n = 4 independent experiments. (**D**–**F**) Immunostaining of NEUROD1 (**D**), TUJ1 (**E**,**F**), DCX (**E**), and MAP2 (**F**) on GBM cells without or with FTT treatment on indicated days. (**G**–**I**) Patch clamp recordings were conducted on GBM cells on day 38 post FTT induction (**G**). Representative traces of action potentials (**H**) or inward sodium currents (**I**) were elicited with injected stepwise currents or voltage. An exemplary trace was highlighted in red. (**J**,**K**) Quantification of purity of neuronal like cells and reprogramming efficiency. n = 3 independent experiments. GBM-3 cells were used in (**B–I**). Data are represented as mean ± SEM. Representative results of n = 3 independent experiments are shown in B and D-I. Scale bar, 50 μm. *p < 0.05; **p < 0.01; ***p < 0.001.

Patient-derived GBM cells could be cultured as adherent monolayer in serum-containing or as sphere in serum-free medium (Fig. 1A). Consistent with previous reports that GBM cells with different culture conditions displayed distinct features^18,19^, CD15^+^, A2B5^+^, SOX2^+^, or NESTIN^+^ cells only existed in serum-free cultured cells, but not in serum cultured cells (Supplementary Fig. S1A,B). Serum cultured cells were positive for astrocytic markers GFAP and S100B, but negative for CD15, A2B5, SOX2, and NESTIN, or neuronal markers MAP2, NEUROD1, and DCX (Supplementary Fig. S1A–D). To exclude the potential inference of CD15^+^, A2B5^+^, SOX2^+^, or NESTIN^+^ cells, serum cultured cells were used to test the neuronal reprogramming capability of different drug combinations.

Previous studies, including ours, showed that human astrocytes were converted into neuronal cells by specific small molecules^11,12^, which included inhibitors for HDAC, TGF-β, Rho kinase, Notch, Shh, BET, and GSK3β signaling pathways. Therefore, we collected approved drugs that act as inhibitors for these pathways (such as Valproic acid as HDAC inhibitor, Tranilast as TGF-β inhibitor, Fasudil as Rho kinase inhibitor, and Tideglusib as GSK3β inhibitor in Supplementary Fig. S2A,B) to induce neuronal reprogramming on GBM cells. As we aimed to repurpose approved drugs, some of the above-mentioned pathways (such as Notch, BET, Shh) were not taken into consideration because there were no approved drugs available to inhibit these signaling pathways. Meanwhile, we also collected Temo, as it showed the capability to reprogram cell fate by converting non-stem glioma cells into stem like cells^20^. Cell morphology and the expression of master neuronal TFs, as well as neuronal marker MAP2, were considered as the primary characteristic properties here to assess neuronal reprogramming^21^, and Brainphys^TM^ medium was used to support neuronal reprogramming and maturation^22^. Temo alone did not induce any neuronal property (Supplementary Fig. S2A–C). The combination of Valproic acid or Tideglusib with Temo did not induce obvious cell morphology change or upregulate neuronal TFs expression either (Supplementary Fig. S2A,B). On the contrary, the combination of Fasudil (an anti-cerebral vasospasm drug) with Temo led to a bipolar morphology on GBM cells (Supplementary Fig. S2A). Meanwhile, the combination of Tranilast (an anti-allergy drug) with Temo upregulated master neuronal TFs such as *MYT1L* and *NEUROD1* (Supplementary Fig. S2B). We then combined Fasudil and Tranilast with Temo, generating a three-drug cocktail (abbreviated as FTT cocktail). Under the treatment of FTT cocktail, neuronal TFs including *ASCL1*, *BRN2*, *MYT1L*, and *NEUROD1* were upregulated (Supplementary Fig. S2C), suggesting that FTT might be capable of inducing neuronal reprogramming on GBM cells.

In order to reveal whether each drug in FTT was essential, we tested neuronal properties on GBM cells treated by any single drug or any two drugs in FTT. Firstly, RT-qPCR analyzing the expression of neuronal TFs showed that Temo alone or any two drugs in FTT cocktail failed to upregulate *ASCL1*, *BRN2*, *MYT1L*, or *NEUROD1* to a comparable level by FTT (Supplementary Fig. S2C). Interestingly, we found that withdrawal of Tranilast from FTT almost completely abolished the capability of FTT to upregulate these TFs. Secondly, under the treatment of FTT, GBM cells exhibited a morphology with rounded cell bodies and long neurite like structure. Removal of any single drug from FTT impaired FTT-induced neuronal like morphology (Supplementary Fig. S2D). Thirdly, the percentage of MAP2^+^ cells in GBM cells treated by any single drug or any two drugs in FTT was significant lower than that by FTT (Supplementary Fig. S2E,F). When Fasudil was removed from FTT, MAP2^+^ cells were undetectable. These data collectively suggested that each drug in FTT cocktail was essential for efficient neuronal reprogramming of GBM cells, thus the three-drug cocktail was used in following studies.

### Reprogramming of serum cultured GBM cells into neuronal like cells by FTT cocktail

Serum cultured cells were digested and cultured in serum medium. On the next day, the medium was changed into Brainphys^TM^-based neuronal induction medium and FTT cocktail or Temo alone was added to treat the cells. FTT cocktail, but not Temo alone, induced cells to change from a flat to a bipolar or multipolar morphology (Fig. 1B). Neuronal TFs including *ASCL1*, *BRN2*, *MYT1L*, *NEUROD1*, and *NEUROG2* were gradually upregulated along FTT treatment (Fig. 1C). Immunostaining also showed that NEUROD1 was positively detected on day 5 cells with FTT treatment (Fig. 1D and Supplementary Fig. S3A). Although cells in control group were positive for TUJ1 (Fig. 1E), they still displayed a flat morphology and were negative for other neuronal markers such as DCX. In contrast, FTT treated cells not only exhibited a bipolar or multipolar morphology, but also were double positive for TUJ1 and DCX on day 5 (Fig. 1E and Supplementary Fig. S3B). On day 8, FTT treated cells were positive for neuronal markers including MAP2, SYN1, and VGLUT1 (Fig. 1F and Supplementary Fig. S3C–E). Furthermore, whole cell patch clamp conducted on day 38 cells showed that action potentials and inward sodium currents were elicited with injected stepwise currents and voltage respectively (Fig. 1G–I), further demonstrating that GBM cells acquired neuronal properties after FTT treatment. Based on MAP2 expression and cell morphology, the purity of neuronal like cells and induction efficiency were about 20–60% and 10–20% respectively (Fig. 1J,K).

### Induction of serum-free cultured GBM cells into neuronal like cells by the same cocktail

**Figure 2:**
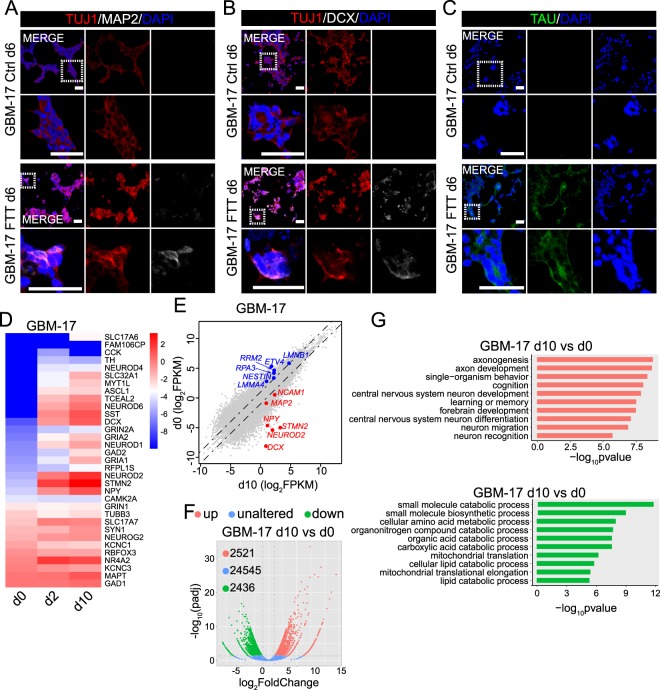
FTT cocktail also induced serum-free cultured GBM cells toward a neuronal fate. (**A–C**) Immunostaining of TUJ1 (**A**,**B**), MAP2 (**A**), DCX (**B**), and TAU (**C**) on GBM-17 cells without or with FTT treatment on day 6. Representative results of n = 3 independent experiments are shown. Scale bar, 50 μm. (**D**) Heatmap showing the expression of neuronal genes on starting GBM cells (d0), and GBM cells with FTT treatment for 2 days (d2) or 10 days (d10). (**E**) Scatter plots comparing gene expression levels between GBM-17 cells with FTT treatment for 10 days (d10) and starting cells (d0). Neuronal genes are highlighted in red and GBM genes in blue. Dashed line indicates a 2-fold change. (**F**) Volcano plots showing the gene expression changes between GBM cells with FTT treatment for 10 days (d10) and starting GBM cells (d0). The dashed vertical and horizontal lines reflect the filter criteria (log_2_(FoldChange) = ±1.0 and *p*adj = 0.05). The upregulated, unaltered, and downregulated genes are highlighted in light red, blue, and green. The numbers in the upper left corner indicate the number of gene with indicated expression changes. (**G**) Gene ontology analysis of the upregulated (highlighted in light red) and downregulated (highlighted in light green) genes in F.

We then tested the effects of FTT on serum-free cultured GBM cells, as these cells closely mirror the phenotype and genotype of a subset of primary tumor cells^18,19^. Similarly, these cells were re-plated and continuing cultured in serum-free medium. On the next day, the cells were treated with FTT and cultured in Brainphys^TM^-based neuronal induction medium. On day 6, except for TUJ1, neuronal markers such as DCX, TAU, or MAP2 were negatively stained on cells without FTT treatment (Fig. 2A–C, and Supplementary Fig. S4A,B). By contrast, TUJ1, DCX, MAP2, and TAU were positively stained on FTT-treated cells on day 6 (Fig. 2A–C, and Supplementary Fig. S4A,B). Using RNA sequencing to analyze the transcriptome of GBM cells without FTT treatment (d0), and GBM cells with FTT treatment for 2 days (d2) or 10 days (d10) showed that FTT treatment dramatically altered the transcriptome of GBM cells (Fig. 2E,F). A panel of neuronal genes, such as *DCX*, *NEURONG2*, *MAP2*, *NEUROD1*, were upregulated with FTT treatment for 2 or 10 days, while tumor associated genes downregulated (Fig. 2D,E). Compared with d0 cells, there were 2,521 upregulated and 2,436 downregulated genes in d10 cells (Fig. 2F). Gene ontology analysis showed that the upregulated genes (log_2_(FoldChange) > 1.0 and padj < 0.05) were highly enriched in neuron development and function (Fig. 2G). These data collectively suggested that FTT turned on the neuronal program in GBM cells.

To test whether CD15^+^ or CD133^+^ cells could be induced into neuronal like cells by FTT, we enriched these cells from serum-free cultured cells by fluorescence activated cell sorting (Supplementary Fig. S4C,E). Using flow cytometry to analyze the purity of enriched cells showed that nearly all cells in enriched CD15^−^ population were negative for CD15 and more than 90% of the cells in CD15^+^ population were positive for CD15 (Supplementary Fig. S4C). The enriched cells were then treated with FTT in above-mentioned neuronal induction medium. TUJ1^+^MAP2^+^ cells were detected in FTT-treated CD15^−^ and CD15^+^ cells on day 6 post treatment (Supplementary Fig. S4D). Similar results were obtained on CD133^−^ and CD133^+^ cells (Supplementary Fig. S4E,F). These results indicated that GBM cells with distinct features might be induced into neuronal like cells by FTT cocktail.

### Regulation of TGF-β, Rho kinase, and CREB signaling might contribute to FTT-mediated neuronal reprogramming of GBM cells

Tranilast was reported to be and has been used for years as a TGF-β inhibitor^13,23^, and Fasudil is a Rho kinase inhibitor. To test whether TGF-β and Rho kinase signaling involved in FTT-induced neuronal reprogramming, we also used some other TGF-β or Rho kinase inhibitors to replace Tranilast or Fasudil in FTT cocktail. Thiazovivin or Y-27632, two Rho kinase inhibitors, were combined with Tranilast and Temo (abbreviated as TTT or YTT, Supplementary Fig. S5) to treat GBM cells. Both TTT and YTT induced GBM cells to exhibit a morphology with rounded cell body and neurite like structure, and express neuronal marker MAP2. The percentage of MAP2^+^ cells by TTT or YTT was comparable to that by FTT (Supplementary Fig. S5A–C). Similarly, when combined with Fasudil and Temo, Repsox or SB431542, two inhibitors for TGF-β, also led to a neuronal morphology and a comparable percentage of MAP2^+^ cells (Supplementary Fig. S5A–C). Previous studies demonstrated that Tranilast also activates Aryl Hydrocarbon Receptor (AHR)^24,25^. Therefore, we also used Leflunomide, an AHR agonist^26^, to replace Tranilast in FTT cocktail. The results showed that when combined with Fasudil and Temo, Leflunomide was much milder than the above mentioned TGF-β inhibitors in inducing neuronal morphology and MAP2^+^ cells (Supplementary Fig. S5A–C). These data suggested that regulation of TGF-β and Rho kinase might contribute to FTT-mediated neuronal reprogramming of GBM cells.

The master neuronal TFs are considered to be the major determinants of neuronal fate decision. Activation of endogenous master neuronal TFs are the key for successful neuronal reprogramming. To explore the potential mechanisms of FTT-mediated neuronal reprogramming, we further analyzed the expression levels of several genes, which were reported to regulate the expression of master neuronal TFs. We found the expression levels of MECP2, a nuclei protein suppressing neuronal gene transcriptional repressors REST^27,28^, and cAMP response element binding (CREB) protein, a transcription factor played important roles in neurogenesis^29,30^, were upregulated, while the expression levels of some other upstream genes such as *PAX6*, *HES1*, *GSK3A*, or *GSK3B* were not significantly altered (Supplementary Fig. S6A,B). We then added U0126 (which abolished the neuronal induction capacity of MECP2^31^) or 666-15 (a potent and selective CREB inhibitor^32^) to treat GBM cells together with the FTT. Unlike U0126, which only attenuated the expression of *BRN2*, 666-15 significantly impaired FTT-induced neuronal like morphology, attenuated FTT-induced upregulation of *ASCL1*, *BRN2*, and *MYT1L*, and reduced the percentage of MAP2^+^ cells (Supplementary Fig. S6C–F), suggesting that CREB signaling might also contribute to  FTT-induced neuronal reprogramming of GBM cells.

### Inhibition of proliferation, promotion of cell death, and attenuation of invasiono on GBM cells by FTT cocktail

**Figure 3:**
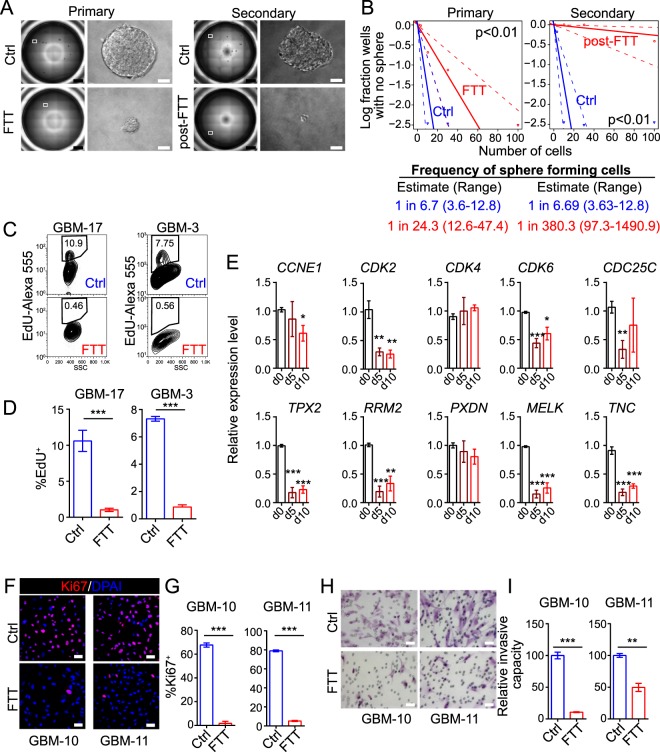
FTT cocktail attenuated malignant properties of GBM cells *in vitro*. (**A**,**B**) Representative results of primary and secondary sphere formation (**A**) and extreme limited dilution assay (**B**) on GBM cells without or with FTT treatment on day 10. A representative well of 96-well plate and a representative sphere are shown in A; scale bar, 1 mm (black), or 50 μm (white). Data in B were analyzed with the online ELDA software and χ^2^ test. Representative results of n = 3 independent experiments are shown. (**C**,**D**) Flow cytometry analysis of EdU incorporation on GBM cells cultured in growth medium without or with FTT treatment for 24 hours. n = 4 and 3 independent experiments for GBM-17 and GBM-3. (**E**) Analysis of cell cycle associated and GBM enriched genes on GBM-10 cells with FTT treatment for 5 (d5) or 10 (d10) days. n = 4 independent experiments. *P* values versus d0 were calculated with two-tailed student’s t test. (**F**,**G**) Immunostaining of Ki67 on GBM cells without or with FTT treatment for 5 days. n = 3 independent experiments. (**H**,**I**) Crystal violet staining of invaded GBM cells without or with FTT treatment for 5 days. n = 3 independent experiments. *p < 0.05; **p < 0.01; ***p < 0.001. Data are represented as mean ± SEM. Two-tailed student’s t test was used to calculate *p* values in (**D**,**G**,**I)**. Scale bar, 50 μm in (**F**,**H)**.

In addition to neuronal properties, we also assessed proliferation, cell death, and invasion on FTT-treated GBM cells. FTT cocktail reduced both the sphere number and size by serum-free cultured GBM cells (Fig. 3A). Extreme limiting dilution assay (ELDA) also showed a lower frequency of sphere forming cells in FTT-treated GBM cells (Fig. 3B). Secondary sphere formation and ELDA showed significant impairment of sphere formation by post-FTT treated cells (Fig. 3A.B). The percentage of EdU^+^ or Ki67^+^ cells in FTT-treated cells were significantly lower (Fig. 3C,D,F,G), suggesting fewer cells were undergoing proliferation in FTT-treated cells. Consistently, the expression levels of cell cycle associated genes (such as *CCNE1*, *CDK2*, *CDK6*, and *CDC25C*) and GBM enriched genes (such as *TPX2*, *RRM2*, *MELK*, and *TNC*), which were highly expressed in GBM cells than normal astrocytes^33^, were downregulated by FTT (Fig. 3E). Temo is an alkylating agent leading to DNA damage. The phosphorylation of histone H2A.X (p-H2A.X) was a marker for DNA damage^34^. We found that Temo treatment increased the percentage of p-H2A.X^+^ cells, but the addition of Tranilast and/or Fasudil to Temo did not further increase p-H2A.X^+^ cells (Supplementary Fig. S7A,B). Even though, FTT cocktail more potently reduced cell viability and promoted cell death than Temo alone (Supplementary Fig. S7C–E), suggesting that Tranilast and Fasudil might potentiate the cytotoxic effect of Temo and sensitize GBM cells to Temo. Transwell invasion assay revealed that the invasive capacity of GBM cells was also attenuated by FTT (Fig. 3H,I). These results collectively demonstrated that FTT inhibited proliferation, promoted cell death, and attenuated invasion on GBM cells.

### Suppression of tumor growth and promotion of survival in GBM PDX by FTT cocktail

**Figure 4:**
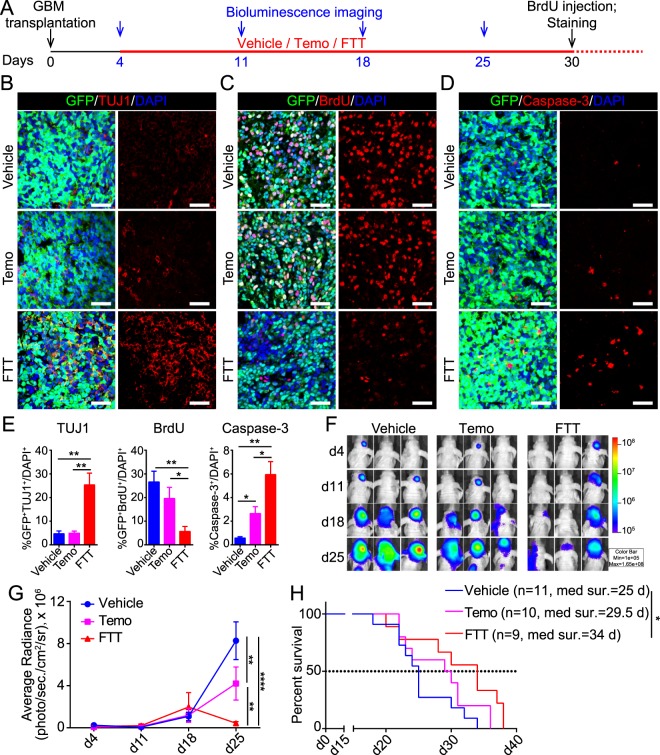
FTT cocktail suppressed tumor growth and prolonged survival in GBM PDX. (**A**) Schematic diagram showing the procedure of *in vivo* experiments. (**B**–**E**) Immunostaining of TUJ1 (**B**), BrdU (**C**), and cleaved Caspase-3 (**D**) on brain sections of mice with indicated treatment. Scale bar, 50 μm. The quantification results are shown in E. (**F**,**G**) Bioluminescence live image monitoring orthotopic tumor growth. Images of 3 representative mice per group are shown in F. Data were statistically analyzed with two-way ANOVA and Tukey’s multiple comparisons test in G. (**H**) Kaplan-Meier survival analysis of GBM-bearing mice with indicated treatment. Log-rank test was used to calculate statistical significances. n = 11, 10, and 9 mice for vehicle, Temo, and FTT group. Data are represented as mean ± SEM. *p < 0.05; **p < 0.01; ***p < 0.001.

GBM cells were infected with lentivirus expressing GFP, so that they could be traced after transplantation into mouse striatum. Four days after transplantation, vehicle, Temo, or FTT was administrated individually (Fig. 4A). Previous studies or the clinical administration routes of these drugs indicated that all the three drugs had a good oral bioavailability and could penetrate the blood brain barrier^2,35–38^. The body weight of each mouse were monitored every two days. Compared to vehicle, Temo alone significantly reduced body weight. The addition of Tranilast and Temo did not further aggravate Temo-induced reduction in body weight (Supplementary Fig. S8A). Similarly, Temo alone significantly reduced the number of Sox2^+^ neural progenitor cells (NPCs) in subventricular zone (SVZ) and the percentage of Ki67^+^ cells in SVZ Sox2^+^ cells (Supplementary Fig. S8B,C), which were consistent with previous studies^39–41^. The addition of Tranilast and Fasudil to Temo did not further exacerbate these effects (Supplementary Fig. S8B,C). Immunostaining of brain sections derived from vehicle mice showed that unlike cultured GBM cells, few GBM cells in the brain were TUJ1^+^ (Fig. 4B,E). There were much more GFP^+^TUJ1^+^ cells in FTT-treated mice, indicating that FTT might also induce GBM cells to acquire neuronal properties *in vivo*. Besides, compared to vehicle or Temo treated mice, FTT treated mice had less BrdU^+^ cells and more cleaved Caspase-3^+^ cells in the tumor (Figs. 4C–E), suggesting FTT cocktail more significantly suppressed proliferation and promoted apoptosis of GBM cells *in vivo*.

To monitor the dynamics of tumor growth, we subcutaneously transplanted GBM cells into the left flank of nude mice and measured the tumor size weekly. Compared with Temo alone, FTT cocktail more significantly inhibited tumor growth (Supplementary Fig. S8D,E). On day 27, when the mice were sacrificed, the tumor weight of FTT-treated mice was lighter than that of Temo- or vehicle-treated mice (Supplementary Fig. S8F). To further monitor the growth dynamics of orthotopically transplanted GBM, we also labeled GBM cells with lentivirus expressing luciferase and orthotopically transplanted them into the brain of nude mice. Tumor growth dynamics was monitored with bioluminescence live imaging. The results showed that FTT cocktail more potently suppressed GBM growth in mouse brain than Temo alone (Fig. 4F,G). Survival analysis revealed that the administration of Temo prolonged the median survival from about 25 days to 29.5 days, and the addition of Tranilast and Fasudil further prolonged to 34 days (Fig. 4H). Statistically, FTT cocktail significantly improved the survival of GBM-bearing mice, while Temo alone did not (Fig. 4H). These data demonstrated that compared with Temo alone, FTT cocktail might more potently suppress GBM growth and promote survival of GBM xenografts.

## Discussion

Bioinformatics analysis of TCGA data revealed that the expression levels of a panel of neuronal genes in GBM were significantly lower than those in non-tumor tissues (Supplementary Fig. S8G), and patients with high *NEUROD1* or *MAP2* expression had a better outcome (Supplementary Fig. S8H). Besides, neuronal reprogramming TFs significantly inhibited tumorigenesis and promoted the survival of GBM mouse model^5–8^. Similarly, the tumorigenicity of B-cell leukemia was impaired with TFs reprogramming leukemia cells into macrophage like cells^9,10^. These data and studies collectively demonstrated that apart from killing tumor cells with conventional cytotoxic agents, cell reprogramming might also be a promising strategy to target malignant tumors. In this study, we identified a FTT cocktail of three approved drugs to reprogram GBM cells into neuronal like cells, potentially providing a promising strategy to improve current treatment for GBM.

Removal of Tranilast or Fasudil from FTT cocktail almost completely abolished the upregulation of neuronal TFs or induced neuronal morphology. Consistently, compared with FTT, any single drug or any two drugs in FTT cocktail failed to achieve a comparable neuronal reprogramming efficiency, suggesting a synergic effect among these drugs and an essential role of each drug, though we are currently not fully aware of the exact role of each drug in inducing neuronal reprogramming. Temo, generally believed as an alkylating agent, reprogrammed non-stem GBM cells into stem like cells^20^, showing its capacity to induce cell reprogramming. Tranilast and Fasudil were inhibitors for TGF-β and Rho kinase, and the effects of Tranilast or Fasudil could be mimicked by some other inhibitors for TGF-β or Rho kinase, suggesting that inhibition of TGF-β and Rho kinase contributed to the neuronal reprogramming of GBM cells. FTT cocktail upregulated the expression of *CREB*. Inhibition of CREB attenuated the neuronal reprogramming capability of FTT, indicating CREB signaling might also contribute to FTT-induced neuronal reprogramming of GBM cells. However, how FTT activated CREB and whether there are some other mediators need to be further investigated. For example, previous study demonstrated that besides cAMP, GSK3 signaling also contributed to neuronal conversion of glioma cells^42^. Though the expression levels of *GSK3A* and *GSK3B* were not significantly altered by FTT (Supplementary Fig. S6A,B), whether GSK3 signaling played a role here awaits further exploration.

With the long-lasting time frame, huge cost, and high failure risk to develop a new drug, repurposing approved drugs provides an efficient alternative strategy to address the unmet medical need^43,44^. Combination of drugs with different molecular mechanisms has shown increased success in drug repurposing and tumor treatment^45–47^. For example, the combination of differentiation-inducing drug all-trans retinoic acid and apoptosis-inducing drug arsenic trioxide brought much better therapy results for acute promyelocytic leukemia than either of the two drugs alone^48,49^. For the three drugs in this study, Temo is the current first-line chemotherapy for GBM. However, the therapeutic effect of Temo is still palliative. Though Tranilast and Fasudil are currently used as non-cancer drugs, it’s possible that these two drugs might benefit glioma treatment, as both of them inhibited the proliferation and invasion of glioma cell lines^50,51^, and Fasudil might also facilitate the targeting of leukemia, pancreatic cancer, and non-small cell lung cancer^52–54^.

Consistent with the *in vitro* experiments, there were more TUJ1^+^ cells in the tumor of FTT-treated mice, indicating that FTT cocktail might also induce neuronal reprogramming of GBM cells *in vivo*. However, the mechanism underlying the tumor suppressive function of FTT might be more complex than merely inducing neuronal reprogramming. Firstly, Temo alone suppressed GBM growth as a DNA alkylating agent. Compared with Temo alone, FTT cocktail induced a comparable DNA damage response on GBM cells (Supplementary Fig. S7A,B). Secondly, Tranilast alone have been reported to inhibit glioma growth by inhibiting TGF-β releasing^50^ and Fasudil increased Temo sensitivity via inhibiting ROCK2/ABCG2^55^. We also found that FTT cocktail more potently inhibited cell proliferation and promoted cell death both *in vitro* and *in vivo* than Temo alone, suggesting that Tranilast and Fasudil might potentiate the cytotoxic effect of Temo and sensitize GBM cells to Temo. Therefore, multiple mechanisms might involve in the tumor suppressive function of FTT. It awaits further exploration to reveal to what extent neuronal reprogramming contributed and the role of each drug.

The introduction of additional side effects was not observed on mouse models after the addition of Tranilast and Fasudil to Temo in this study. For example, the body weight of FTT-treated mice was comparable to that of Temo alone treated mice. The addition of Tranilast and Fasudil did not exacerbate Temo-induced reduction in the number and proliferation of resident SVZ NPCs in mouse brain either. However, the long-term and more detailed *in vivo* effects of FTT should be closely monitored in future. For example, whether FTT affects the survival and proliferation of resident stem cells in other tissues and the functionality of other organs. Besides, though it seemed that the majority of glioma-derived neuronal like cells could not survive *in vivo*^7^, which might avoid introducing abnormal neural circuits. Whether FTT-induced neuronal like cells would survive, become mature, or even interfere neural activity also need further investigation.

Taken together, the FTT cocktail identified here reprogramed GBM cells into neuronal like cells and more potently suppressed GBM growth in PDX than Temo alone. Though the mechanisms underlying FTT-mediated neuronal reprogramming and tumor suppression were not fully understood, the drug cocktail identified in a cell reprogramming logic and the neuronal reprogramming by the drug cocktail might improve the current treatment for GBM.

## Methods

### Human specimen and animals

The collection of GBM surgical specimens was approved by the Institutional Review Board of Huashan Hospital, Fudan University, and the informed consents were obtained from patients or their guardians. We have complied with all relevant ethical regulations for the collection of these specimens. The used specimens were classified as GBM by neuropathologist according to the WHO classification system (Supplementary Table S1). All the animal experiments were performed in accordance with the NIH Guide for the Care and Use of Laboratory Animals and approved by the Institutional Animal Care and Use Committee (IACUC) of Shanghai Institute of Biochemistry and Cell Biology, Chinese Academy of Sciences. Athymic BALB/c nude mice were purchased from Shanghai SLAC Laboratory Animal Co., Ltd. (Shanghai, China) and housed in 12 hours light/dark cycle and allowed *ad libitum* access to water and food. Efforts were made to reduce the number of used mice and to minimize pain and suffering.

### Isolation and culture of GBM cells

GBM specimen immersed in Neurobasal medium (Gibco) was placed in ice and transferred to the lab within 1 hour after resection. The specimen was adequately washed by D-Hank’s solution to remove the blood and then minced in a petri dish on ice by a scissor. For serum cultured GBM cells, minced tissue was digested with 0.125% trypsin in the 37 °C incubator for 25 minutes. The petri dish containing tissue and trypsin was shaken every 5 minutes. After digestion, DMEM with 10% FBS was used to stop the digestion. The tissue was triturated into single cells by a 5 ml pipette and then filtered by a 40 μm filter to remove debris. After centrifugation, the suspension was aspirated and red blood cells were removed by 1 ml RBC lysis buffer (1 mM KHCO_3_, 150 mM NH_4_Cl, and 0.1 mM EDTA-Na_2_) at room temperature. Five minutes later, 10 ml DMEM with 10% FBS was added in and cells was centrifuged. Cell pellet was re-suspended with and cultured in serum medium (DMEM with 10% FBS). For subculture, cells were passaged by trypsin once they reached 80–90% confluence. Experiments were performed on cells within passage 2 and 5 because of the limited passages and to minimize genetic drift. For isolation of serum-free cultured GBM cells, minced specimen was digested with accutase (Gibco) for 25 minutes at 37 °C in a petri dish, which was shaken every 5 minutes. After digestion, the digested tissue was transferred into a 50 ml falcon tube containing 10 ml DMEM/F12 (Gibco), triturated into single cells by a 5 ml pipette, and filtered by a 40 μm filter to remove tissue debris. After centrifugation, cell pellet was re-suspended with 1 ml RBC lysis buffer to remove red blood cells as mentioned above. Single cells were plated into petri dishes, which were pretreated with 10 ug/ml laminin (Sigma) for 2 hours at 37 °C, and cultured in serum-free medium, which was consisted of Neurobasal medium (Gibco), 1X N2 (Gibco), 1X B27 (Gibco), 1X non-essential amino acid (Millipore), 1X sodium pyruvate (Sigma), 1X glutamax (Gibco), and supplemented with 20 ng/ml basic fibroblast growth factor (bFGF, Invitrogen), 20 ng/ml epidermal growth factor (EGF, Invitrogen). After the cells reached a confluence above 50%, cells were digested by accutase and then maintained as sphere in low attachment dishes. Spheres were digested into single cells by accutase every three to four days once the diameter of majority spheres reached 100 µm. Growth factors including bFGF and EGF were supplemented into the culture medium every day at a final concentration of 10 ng/ml for each factor.

GBM-3, GBM-4, GBM-10, GBM-11, GBM-17, and GBM-25 cells were isolated from patient samples in this study. TJ-17 cells in Supplementary Fig. S4 were generated and kindly provided by Dr. Zhengliang Gao (Tongji University). The detailed pathological information for these tissue samples were listed in Supplementary Table S1. Nomenclature of GBM cells follows the structure GBM-XX, where XX was a unique identifier of patient sample. GBM-3, GBM-4, GBM-10, GBM-11, and GBM-25 cells were cultured as monolayer in serum medium. Due to the limited passages of serum cultured GBM cells and to avoid genetic drift, serum cultured cells at low passages (<5 passages) were used to conduct indicated experiments. GBM-17 and TJ-17 cells were cultured in serum-free medium. STR fingerprint for GBM-3 is: X/Y (AMEL), 10/11 (D5S818), 8/12 (D13S317), 11/14 (D7S820), 12/12 (D16S539), 14/17 (vWA), 9/9 (TH01), 8/8 (TPOX), 10/11(CSF1PO1); for GBM-4 is: X/X (AMEL), 11/13 (D5S818), 8/12 (D13S317), 9/11 (D7S820), 11/13 (D16S539), 17/17 (vWA), 7/10 (TH01), 8/11 (TPOX), 10/12 (CSF1PO1); for GBM-17 is: X/X (AMEL), 11/11 (D5S818), –/–(D13S317), 10/11 (D7S820), 11/13 (D16S539), 14/16 (vWA), 8/8 (TH01), 8/11 (TPOX), 11/11 (CSF1PO1). Cell cultures were regularly tested to be free of mycoplasma contamination.

### Induction of neuronal like cells on GBM cells

For induction on serum cultured cells, cells were plated onto corning costar plates without or with coverslips (pretreated with 0.1 mg/ml poly-D-lysine (Sigma) for 2 hours at 37 °C) at a density of 50,000 cells per square centimeter in DMEM with 10% FBS. After 24 hours, the confluence should be above 90% and the medium was replaced by neuronal induction medium, which was BrainPhys neuronal medium (Stemcell Technologies) supplemented with 1X B27, 1X N2, 10 ng/ml brain-derived neurotrophic factor (Peprotech), 10 ng/ml glial cell-derived neurotrophic factor (Peprotech), 10 ng/ml insulin-like growth factor (Peprotech), 0.2 μM ascorbic acid (Sigma), and 100 μM N6, 2′-O-Dibutyryladenosine 3′,5′-cyclic monophosphate sodium salt (Sigma). FTT cocktail (50 μM Temo (Selleck), 100 μM Tranilast (TCI Chemicals), and 50 μM Fasudil (Selleck)) was added as indicated. The neuronal induction medium without or with FTT was changed every four days. For induction on serum-free cultured cells and post-sorted cells, cells were cultured as monolayer by plating onto corning costar plates without or with coverslips (pre-coated with 0.1 mg/ml poly-D-lysine for 2 hours at 37 °C and then with 10 ug/ml laminin for 2 hours at 37 °C) at a density of 50,000 cells per square centimeter in serum-free medium supplemented with 1 μg/ml laminin. On the next day, the medium was changed into neuronal induction medium containing 1 μg/ml laminin without or with FTT as indicated. The neuronal induction medium containing laminin without or with FTT was changed every four days. Brightfield images of indicated samples were captured with Zeiss Observer. Z1 microscope. Valproic acid (VPA) (Sigma, final concentration at 0.5 mM) and Tideglusib (Selleck, final concentration at 30 μM) were used during the identification of neuronal inducing drug cocktail. 1 μM Repsox (Selleck), 5 μM SB431542 (MedchemExpress), 5 μM Y-27632 (Selleck), 5 μM Thiazovivin (MedchemExpress), and 50 μM Leflunomide (Selleck) were used to replace Tranilast or Fasudil. 10 μM U0126 (MedChemExpress) and 0.5 μM 666-15 (MedChemExpress) were used to explore the mechanism.

### RNA isolation and quantitative real-time PCR

RNA isolation and quantitative real-time PCR were performed as briefly described^11^. Briefly, total RNA of indicated samples was isolated with Trizol Reagent (Sigma) following manufacturer’s instructions. 1 μg RNA was used for reverse transcription with M-MLV reverse transcriptase (Promega) and random hexamers according to manufacturer’s instructions. Quantitative real-time PCR was conducted with gene specific primers and 2x HotStart SYBR Green qPCR Master Mix (Excell, Cat. #MB000-3013) in an MX3000P Stratagene PCR machine. The relative expression levels were normalized to the internal control (HPRT). Primers used for qPCR are listed in Supplementary Table S2.

### RNA sequencing and data analysis

Total RNA of GBM cells was purified as mentioned above. RNA sequencing and data analysis were performed by Beijing Novogene Bioinformatics Technology Co., Ltd. RNA quantification and qualification were performed with NanoPhotometer spectrophotometer, Qubit RNA Assay Kit in Qubit 2.0 Flurometer (Life Technologies), and the RNA Nano 6000 Assay Kit of the Bioanalyzer 2100 system (Agilent Technologies). A total amount of 3 μg RNA per sample was used as input material for the RNA sample preparations. Sequencing libraries were generated using NEBNext UltraTM RNA Library Prep Kit for Illumina (NEB) following manufacturer’s recommendations. The clustering of the index-coded samples was performed on a cBot Cluster Generation System using TruSeq PE Cluster Kit v3-cBot-HS (Illumia) according to the manufacturer’s instructions. After cluster generation, the library preparations were sequenced on an Illumina Hiseq platform and 150 bp paired-end reads were generated. Clean data (clean reads) were obtained by removing reads containing adapter, reads containing ploy-N and low quality reads from raw data. Index of the reference genome (GRCh38 (hg38)) was built using Hisat2 v2.0.5 and paired-end clean reads were aligned to the reference genome using Hisat2 v2.0.5. The featureCounts v1.5.0-p3 was used to count the reads numbers mapped to each gene. And then FPKM of each gene was calculated based on the length of the gene and reads count mapped to this gene. Prior to differential gene expression analysis, for each sequenced library, the read counts were adjusted by edgeR program package through one scaling normalized factor. Differential expression analysis of two conditions was performed using the edgeR R package (3.18.1). The *p* values were adjusted using the Benjamini & Hochberg method. Corrected p-value of 0.05 and absolute foldchange of 2 were set as the threshold for significantly differential expression. Gene Ontology (GO) enrichment analysis of differentially expressed genes was implemented by the clusterProfiler R package, in which gene length bias was corrected. GO terms with corrected *p* value less than 0.05 were considered significantly enriched by differential expressed genes.

### Cell viability measurement

Cell viability was measured by CellTiter-Glo reagent (Promega) following manufacturer’s instructions. Briefly, GBM cells were plated into 96-well plates at a density of 5,000 cell per well with GBM growth medium. The medium was changed into neuronal induction medium with indicated drug(s) 24 hours later. After aspiration of the medium, 1X CellTiter-Glo reagent was added into the wells to measure the cell viability on indicated days. After mixed for 2 minutes on an orbital shaker and incubated for 10 minutes at room temperature, the reagents were transferred into an opaque-walled 96-well plates and luminescence was recorded with SpectraMax M5 and SoftMax Pro Software (v5.4.1, Molecular Devices).

### Flow cytometry and fluorescence activated cell sorting (FACS)

Cells for flow cytometry analysis or FACS were digested into single cells with accutase and washed once with cold MACS buffer (0.5% FBS and 2 mM EDTA in PBS). For cell surface protein (CD15, A2B5, or CD133) staining, cells were incubated with indicated primary or matched isotype control antibody (diluted with MACS buffer) for 30 minutes on ice. After washed with MACS buffer for twice, fluorochrome conjugated secondary antibody (diluted with MACS buffer) was added and incubated on ice in dark for 30 minutes. For intracellular staining of p-H2A.X, cells were fixed and permeabilized for 30 minutes on ice with fixation/permeabilization buffer (eBioscience) and then washed once with permeabilization buffer (eBioscience). Indicated primary or matched isotype control antibody was diluted with permeabilization buffer and incubated for 30 minutes on ice. After washed with permeabilization buffer for twice, fluorochrome conjugated secondary antibody (diluted with permeabilization buffer) was added and incubated on ice in dark for 30 minutes. After incubated with secondary antibody and then washed with indicated wash buffer (MACS buffer for surface staining and permeabilization buffer for intracellular staining), the cells were ready for analysis or sorting. BD LSRII and BD Influx were used for flow cytometry analysis and FACS respectively. To evaluate the purity of post-sorted cells, we analyzed post-sorted and pre-sorted cells simultaneously by flow cytometry right after sorting. Data were analyzed with Flowjo software. The primary antibodies used were: CD15 (Santa Cruz, Cat. #sc-21702), A2B5 (Miltenyi Biotec, Cat. #130-093-394), CD133 (Miltenyi Biotec, Cat. #130-090-422), and phospho-Histone H2A.X (Ser 139) (Cell Signaling Technology, Cat. #9718). The following secondary antibodies were used: Goat-anti-mouse-FITC (Invitrogen, Cat. #A-10679) and Donkey-anti-rabbit-Alexa 488 (Molecular Probes, Cat. #A21206). The used antibodies were validated by manufacturer, in previous studies and/or in our experiments.

### Immunofluorescence staining

Immunofluorescence staining was performed as previously described^11^. Briefly, for cultured cells, after washed with PBS, cells cultured on coverslips were fixed with 4% paraformaldehyde (PFA) at room temperature for 10 minutes. Afterwards, cells were washed with PBS for twice and then permeabilized and blocked with permeabilization and blocking buffer (1% BSA and 0.5% Triton X-100 in PBS) at room temperature for 1 hour. Indicated primary antibodies (diluted with permeabilization and blocking buffer) were incubated at 4 °C overnight. On the next day, after adequate wash with PBS, cells were incubated with fluorochrome conjugated secondary antibodies (diluted with permeabilization and blocking buffer) for 1 hour at room temperature. Nuclei was stained with DAPI (Beyotime) at room temperature for 5 minutes. The coverslip was mounted with Mowiol and then left to dry at 4 °C. Purity of neuronal-like cells and reprogramming efficiency were calculated as previously described^11^.

The brains for immunostaining were harvested from fresh post-mortem mice or mice sacrificed on day 30. For preparation of brain sections, mice were anesthetized and perfused with PBS (20 ml per mouse) and then 4% PFA (10 ml per mouse). Brains were collected and fixed in 4% PFA (10 ml per brain) overnight at 4 °C. On the next day, fixed brains were washed with PBS for three times and then dehydrated with 30% sucrose (in PBS, 10 ml per brain) for 48 hours at 4 °C. After brains settled to the bottom of falcon tubes, they were transferred into frozen section medium (Thermo Fisher Scientific, Cat. #6502) on the cryostat chuck. Brains were frozen sectioned at 30 μm thickness. The sections were preserved at −80 °C for further staining. For immunofluorescent staining of brain sections, sections were washed with PBS for once and then permeabilized and blocked with permeabilization and blocking buffered as mentioned above. The following procedure was the same as that on cultured cells. For staining of TUJ1 on brain sections, antigen retrieval was performed with antigen retrieval solution (Beyotime, Cat. #P0090) following manufacturer’s instructions before permeabilization and blocking. For BrdU staining, sections were treated in 1 M HCl at 37 °C for 30 minutes to denature DNA and then rinsed with PBS before permeabilization and blocking.

For staining of sphere sections, the spheres were allowed to settle down by gravity in a 15 ml falcon tube and then the culture medium was aspirated. Spheres were fixed with 4% PFA at room temperature for 15 minutes. Afterwards, the spheres were dehydrated as mentioned above. The following procedure was the same as that for brain sections.

The primary antibodies used were NESTIN (1:1000, Millipore, Cat. #MAB5326), SOX2 (1:250, Santa Cruz, Cat. #sc-17320), NEUROD1 (1:500, Abcam, Cat. #AB60704), TUJ1 (1:500 for cultured cells, 1:250 for brain sections, Biolegend, Cat. #801202), DCX (1:200, Santa Cruz, Cat. #sc-8066), MAP2 (1:500 for cultured cells, 1:250 for brain sections, Millipore, Cat. #AB5622), Ki67 (1:500, Abcam, Cat. #ab15580), BrdU (1:250, AbD Serotec, Cat. #OBT0030G), Cleaved Caspase-3 (1:200, Cell signaling technology, Cat. #9661), GFAP (1:1000, DAKO, Cat. #Z033401), S100B (1:250, Sigma, Cat. #S2532), SYN1 (1:500, Millipore, Cat. #AB1543), VGLUT1 (1:500, Synaptic system, Cat. #135302), and TAU (1:200, Invitrogen, Cat. #13-6400). The following secondary antibodies were used: Donkey-anti-rat-cy3 (Jackson ImmunoResearch, Cat. #712-165-150), Donkey-anti-goat-Alexa 488 (Molecular Probes, Cat. #A11055), Donkey-anti-mouse-Alexa 488 (Molecular Probes, Cat. #A21202), Donkey-anti-rabbit-Alexa 488 (Molecular Probes, Cat. #A21206), Donkey-anti-goat-cy3 (Jackson ImmunoResearch, Cat. #705-165-147), Donkey-anti-rabbit-cy3 (Jackson ImmunoResearch, Cat. #711-165-152), Donkey-anti-mouse-cy3 (Jackson ImmunoResearch, Cat. #715-165-150). These antibodies were widely used in the field and are also validated by the vendor and/or in previous publications. Images were captured and analyzed with Leica SP8 microscope at National Center for Protein Science Shanghai.

### Electrophysiology detection

GBM cells were induced into neuronal like cells as mentioned above. 16 days after induction, cells were cultured in neuronal induction medium without further drug cocktail treatment. Electrophysiological analysis on indicated cells was performed as previously described^11,16^. Briefly, whole-cell recordings were performed using Multiclamp 700B amplifier (Molecular Devices). The bath was constantly perfused with ACSF, bubbled with 95% O2/5% CO2. The pipette solution contained (in mM) 93 K-gluconate, 16 KCl, 2 MgCl_2_, 10 HEPES, 4 ATP-Mg, 0.3 GTP-Na_2_, 10 Na-phosphatecreatine, 0.5 Alexa Fluor 488 (Invitrogen), and 0.4% neurobiotin (Invitrogen) (pH 7.25, 290–300 mOsm). Membrane potentials were hold around −20 mV, and step currents with an increment of 10 pA were injected to elicit action potentials. Step voltages with an increment of 10 mV were injected to elicit inward sodium current. Signals were sampled at 5 kHz with a 2 kHz low-pass filter. Recordings with Ra > 50 MΩ or fluctuation >20% were excluded. Data were analyzed with pClamp 10 software (Clampfit).

### Cell invasion assay

Transwell plates (Corning, Cat. #3422) were used to analyze cell invasive capacity. The bottom side of the inserted membrane was coated with 0.1% gelatin (Sigma) for 30 minutes at 37 °C. Cells treated without or with FTT treatment in neuronal induction medium for 5 days were digested into single cells. 20,000 single cells were seeded on the top side of the membrane with 100 μl serum-free DMEM/F12 medium. 500 μl DMEM/F12 medium containing 10% FBS was added into the well of the plate (the bottom side of the membrane). After cultured in 5% CO_2_ at 37 °C for 12 hours, the cells were fixed by 4% PFA and then stained with crystal violet solution. After staining, the non-invasive cells at the upper side of the membrane were scraped off with cotton swabs. Invasive cells in 10 random fields were counted under a light microscope. The representative images were captured with Zeiss Observer. Z1 microscope.

### EdU incorporation assay

For EdU incorporation, cells were cultured in growth medium without or with FTT treatment. 24 hours later, 10 μM EdU (Invitrogen, Cat. #C10353) was added into the medium. After incubation at 37 °C for 2 hours, the cells were digested into single cells with accutase and then fixed with 4% PFA at room temperature for 15 minutes. After wash with PBS for once, the cells were permeabilized with permeabilization buffer (PBS with 0.1% saponin). 10 minutes later, click-iT plus reaction mixture (Invitrogen, Cat. #C10353) was added in and then the cells were incubated at room temperature for 30 minutes in dark. After washed once with permeabilization buffer, cells were re-suspended with PBS and were ready for flow cytometry analysis. BD LSRII and Flowjo software were used for data acquisition and analysis respectively.

### Sphere formation and extreme limiting dilution assay

Sphere formation was performed by plating 100 single cells into each well of 96-well plate in serum-free medium without or with FTT cocktail. For secondary sphere formation, GBM cells in serum-free medium without or with FTT treatment for 6 days were digested into single cells. 100 single cells were plated into per well of 96-well plate and cultured in serum-free medium without further FTT treatment. The images of per well were captured 10 days later using Zeiss Observer. Z1 microscope and stitched with stitching plugin in ImageJ.

Extreme limiting dilution assay was performed as previously described with slightly modification^56,57^. Briefly, GBM sphere was dissociated into single cells and plated into 96-well plate at a density of 1, 3, 10, 30, or 100 cells per well in serum-free medium without or with FTT treatment. Wells without or with sphere were counted 10 days later and analyzed with the online software (http://bioinf.wehi.edu.au/software/elda/)^58^. For secondary extreme limiting dilution assay, GBM spheres in serum-free medium without or with FTT treatment for 6 days were digested into single cells. Single cells were plated into the wells of 96-well plate with 1, 3, 10, 30, or 100 cells per well and cultured in serum-free medium without further FTT treatment. Wells without or with sphere were counted 10 days later and analyzed with the online ELDA software as mentioned above.

### Orthotopic and subcutaneous xenografts

Female BALB/c nude mice of 6 weeks old were used as xenograft recipient. Primary GBM cells were cultured in serum-free medium and labeled with GFP and luciferase expressing lentivirus, which was prepared as previously reported^11^. For intracranial transplantation, cells were digested into single cells by accutase and suspended with cold PBS at a density of 1 × 10^5^ cells/μl. Cell suspension was placed on ice and tapped occasionally. Mice were anesthetized with pentobarbital sodium and placed into a stereotaxic apparatus. 5 × 10^5^cells (5 μl cell suspension) were injected into the striatum by a Hamilton needle (Hamilton, Cat. #701 N) at the following coordinate (relative to Bregma, in mm): AP: +1.0; ML: +2.0; DV: −2.5. The infusion rate was 1 μl/minute and was controlled by a digital pump. After infusion, the needle was kept in place for additional 2 minutes and then slowly withdrawn to minimize the backflow of injected cells. Mice were returned to the housing cages after full recovery from anesthesia. Because of ethical reasons, mice were sacrificed when the loss of body weight ≥20% or when mice had trouble in drinking, feeding, or ambulating. Based on previous experience for bioluminescence imaging and survival analysis on GBM-bearing mice, sample size typically of about 8 mice was sufficient to ensure reproducibility. The experiments for bioluminescence imaging and survival analysis were performed with at least 8 mice per group. For survival analysis, mice were allowed to natural death and the dates when mice died or were sacrificed because of ethical reasons were recorded. For subcutaneous transplantation, 2 × 10^6^ cells were subcutaneously transplanted into the left flank. Tumor size (length and wide) was measured using caliper at indicated time. Tumor volume was calculated using the formula V = (π/6) *L*W^2^, where L and W indicate tumor’s length and width respectively. GBM-17 cells were used for animal experiments.

### Bioluminescence imaging

Bioluminescent imaging was performed on indicated days with IVIS Spectrum *in vivo* imaging system (Xenogen) to monitor the tumor growth dynamics. D-luciferin (Shanghai Biochempartner Co., Ltd) was intraperitoneally injected at 150 mg/kg body weight and then mice were placed under isoflurane anesthesia. Images were captured 10 minutes after injection with the following parameters: Luminescent image: Exposure Time, 1 minutes; Binning, Medium; f/stop, 1; Excitation Filter, Block; Emission Filter, Open; Photographic image: Exposure time, 0.2 minutes; Binning, Medium; f/stop, 8. When analyzing of tumor growth dynamics from day 0 to day 25 (Fig. 4), we excluded the mice that had died before day 25, as the bioluminescence data for these mice on day 25 were not available. This criteria was pre-established.

### Drug treatment and BrdU labeling

Mice were randomized based on the result of bioluminescence imaging on day 4 by a blinded investigator so that each group contained an equivalent range of tumor size. Drugs were administrated daily since day 4 till death. 0.5% sodium carboxymethylcellulose (Sigma) was used as vehicle. Temo and Fasudil for animal experiments were purchased from Shanghai Biochempartner Co., Ltd, and Tranilast was purified from Tranilast capsules (China Pharmaceutical University Pharmaceutical Company). Mouse weight was monitored every two days. Temo, Tranilast, and Fasudil were orally gavaged once per day till death at 30, 120, and 60 mg/kg body weight. For BrdU labeling, BrdU (Sigma) was intraperitoneally injected at 50 mg/kg body weight on day 30. Mice were sacrificed 1 hour later to harvest brains for further BrdU staining.

### Statistical Analysis

All quantified data were presented as average ± SEM. Unless specified in the figure legends, two-tailed student’s t tests were used to calculate statistical significance with *p* values between two groups. For comparison among three or more groups, one-way or two-way ANOVA and Tukey’s multiple comparisons test were applied. For survival curves analysis, Kaplan-Meier survival curves were made by GraphPad Prism 6 and analyzed by Log-rank analysis. ELDA results were analyzed with the ELDA online software (http://bioinf.wehi.edu.au/software/elda/)^58^ and chisq test in the software was used to calculate the statistical differences. A *p* value < 0.05 was considered as statistically significant.

### Accession numbers

The accession number for the RNA-seq dataset in this study is GEO: GSE110130.

## Supplementary information

Supplementary figures and tables

## Data Availability

Data supporting the findings of this study are included within the article and from the corresponding author upon reasonable request.
