# Author Correction: Suppression of glioblastoma by a drug cocktail reprogramming tumor cells into neuronal like cells

**DOI:** 10.1038/s41598-020-59777-8

**Published:** 2020-02-14

**Authors:** Longfei Gao, Shichao Huang, Hong Zhang, Wei Hua, Shunmei Xin, Lin Cheng, Wuqiang Guan, Yongchun Yu, Ying Mao, Gang Pei

**Affiliations:** 1 State Key Laboratory of Cell Biology, CAS Center for Excellence in Molecular Cell Science, Shanghai Institute of Biochemistry and Cell Biology, Chinese Academy of Sciences, University of Chinese Academy of Sciences, 320 Yueyang Road, Shanghai, 200031 China; 2 Shanghai Key Laboratory of Signaling and Disease Research, Collaborative Innovation Center for Brain Science, School of Life Sciences and Technology, Tongji University, Shanghai, 200092 China; 3 School of Life Science and Technology, ShanghaiTech University, 100 Haike Road, Shanghai, 201210 China; 4 Department of Neurosurgery, Huashan Hospital, Fudan University, 12 Middle Wulumuqi Road, Shanghai, 200040 China; 5 State Key Laboratory of Medical Genomics, Shanghai Institute of Hematology, Rui Jin Hospital, Shanghai Jiao Tong University School of Medicine, Shanghai, 200025 China; 6 Institute of Neurobiology, Institutes of Brain Science, State Key Laboratory of Medical Neurobiology and Collaborative Innovation Center for Brain Science, Fudan University, Shanghai, 200032 China; 7 State Key Laboratory of Medical Neurobiology, School of Basic Medical Sciences, Institutes of Brain Science and Collaborative Innovation Center for Brain Science, Fudan University, 131 Dong’an Road, Shanghai, 200032 China; 8 Institute for Stem Cell and Regeneration, Chinese Academy of Sciences, Beijing, 100101 China

Correction to: *Scientific Reports* 10.1038/s41598-019-39852-5, published online 05 March 2019

In the Supplementary Information file originally published with this Article, an error was made during the assembly of Figure S1. In this figure, immunostaining was performed to show that serum cultured GBM cells were negative for neural progenitor markers NESTIN and SOX2 (Figure S1B), and also negative for neuronal markers such as NEUROD1 and DCX (Figure S1C). The authors noticed that the representative image showing NESTIN and SOX2 staining on serum cultured cells in Figure S1B was identical to the image showing NEUROD1 and DCX staining on GBM-3 cells in Figure S1C in the initial file. After carefully checking the original data, the authors realized that the image in Figure S1B is correct, whereas the Figure S1C in the initial file was incorrect owing to erroneous assembly.

This error has been corrected in the Supplementary Information that now accompanies the Article.

