# Supplementary material for: Suppression of glioblastoma by a drug cocktail reprogramming tumor cells into neuronal like cells
Source: Sci Rep. 2019 Mar 5;9:3462. doi: 10.1038/s41598-019-39852-5 (PMC6401026; doi:10.1038/s41598-019-39852-5)
Supplement: Supplementary file 1 — Supplementary figures and tables [file 41598_2019_39852_MOESM1_ESM.pdf]

Suppression of glioblastoma by a drug cocktail reprogramming tumor cells into neuronal like cells

Longfei Gao[1, 8], Shichao Huang[2, 8], Hong Zhang[1, 3], Wei Hua[4], Shunmei Xin[1], Lin Cheng[5], Wuqiang Guan[6], Yongchun Yu[6], Ying Mao[4, 7], and Gang Pei[1, 2, *]

[1]State Key Laboratory of Cell Biology, CAS Center for Excellence in Molecular Cell Science, Shanghai Institute of Biochemistry and Cell Biology, Chinese Academy of Sciences; University of Chinese Academy of Sciences, 320 Yueyang Road, Shanghai 200031, China
[2]Shanghai Key Laboratory of Signaling and Disease Research, Collaborative Innovation Center for Brain Science, School of Life Sciences and Technology, Tongji University, Shanghai 200092, China
[3]School of Life Science and Technology, ShanghaiTech University, 100 Haike Road, Shanghai 201210, China
[4]Department of Neurosurgery, Huashan Hospital, Fudan University, 12 Middle Wulumuqi Road, Shanghai 200040, China
[5]State Key Laboratory of Medical Genomics, Shanghai Institute of Hematology, Rui Jin Hospital, Shanghai Jiao Tong University School of Medicine, Shanghai 200025, China
[6]Institute of Neurobiology, Institutes of Brain Science, State Key Laboratory of Medical Neurobiology and Collaborative Innovation Center for Brain Science, Fudan University, Shanghai 200032, China
[7]State Key Laboratory of Medical Neurobiology, School of Basic Medical Sciences, Institutes of Brain Science and Collaborative Innovation Center for Brain Science, Fudan University, 131 Dong'an Road, Shanghai 200032, China

[8] Longfei Gao and Shichao Huang contributed equally to this work.

*Correspondence: gpei@sibs.ac.cn

Supplementary Information

Supplementary Figures

Figure S1. Characterization of cultured GBM cells.

Figure S2. Identification of the neuronal reprogramming drug cocktail FTT.

Figure S3. Reprogramming of serum cultured GBM cells into neuronal like cells by FTT.

Figure S4. Induction of serum-free cultured TJ-17 cells into neuronal like cells by FTT.

Figure S5. TGFβ and Rho kinase inhibition in neuronal reprogramming of GBM cells.

Figure S6. CREB signaling in FTT-mediated neuronal reprogramming.

Figure S7. Further analysis of FTT on GBM cells.

Figure S8. The effects of FTT cocktail in vivo and expression of neuronal genes in GBM patients.

Supplementary Tables

Table S1. Specimen used in this study

Table S2. RT-qPCR primers used in this study

Supplementary Figures

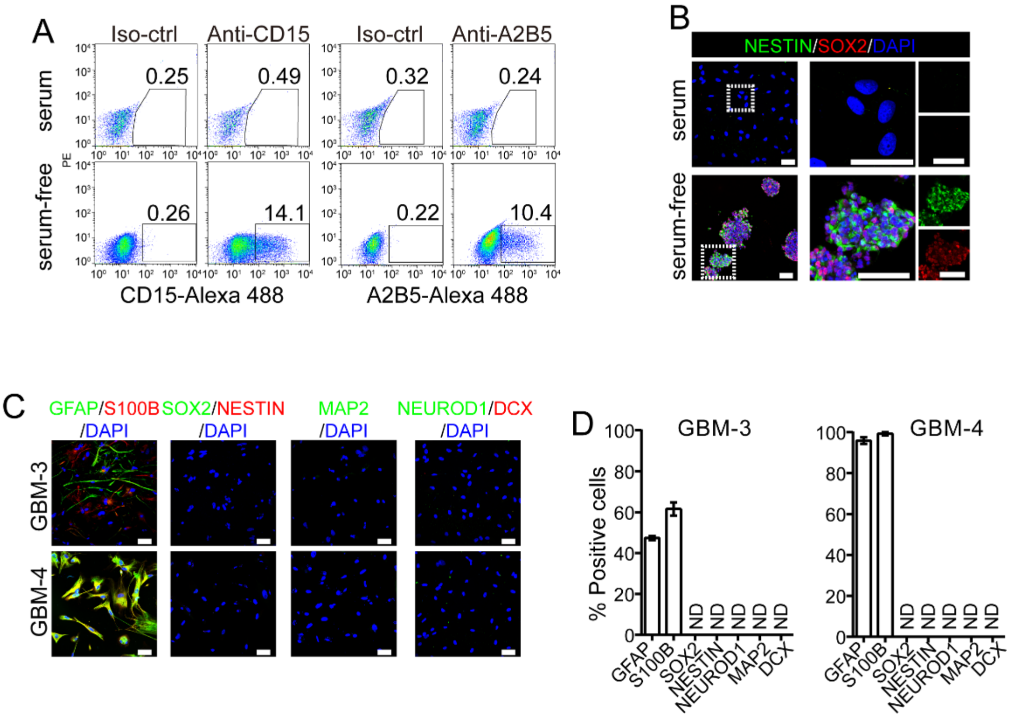

Figure S1. Characterization of cultured GBM cells.

(**A**-**B**) Detection of CD15, A2B5, NESTIN, and SOX2 on serum or serum-free cultured GBM cells. Representative results of n=3 independent experiments are shown. Scale bar, 50 μm.

(**C**-**D**) Immunostaining of astrocyte genes (GFAP, S100B), stem cell genes (SOX2, NESTIN), and neuronal genes (MAP2, NEUROD1, DCX) on serum cultured GBM cells. ND, not detectable. n=3 independent experiments. Scale bar, 50 μm. Data are represented as mean ± SEM.

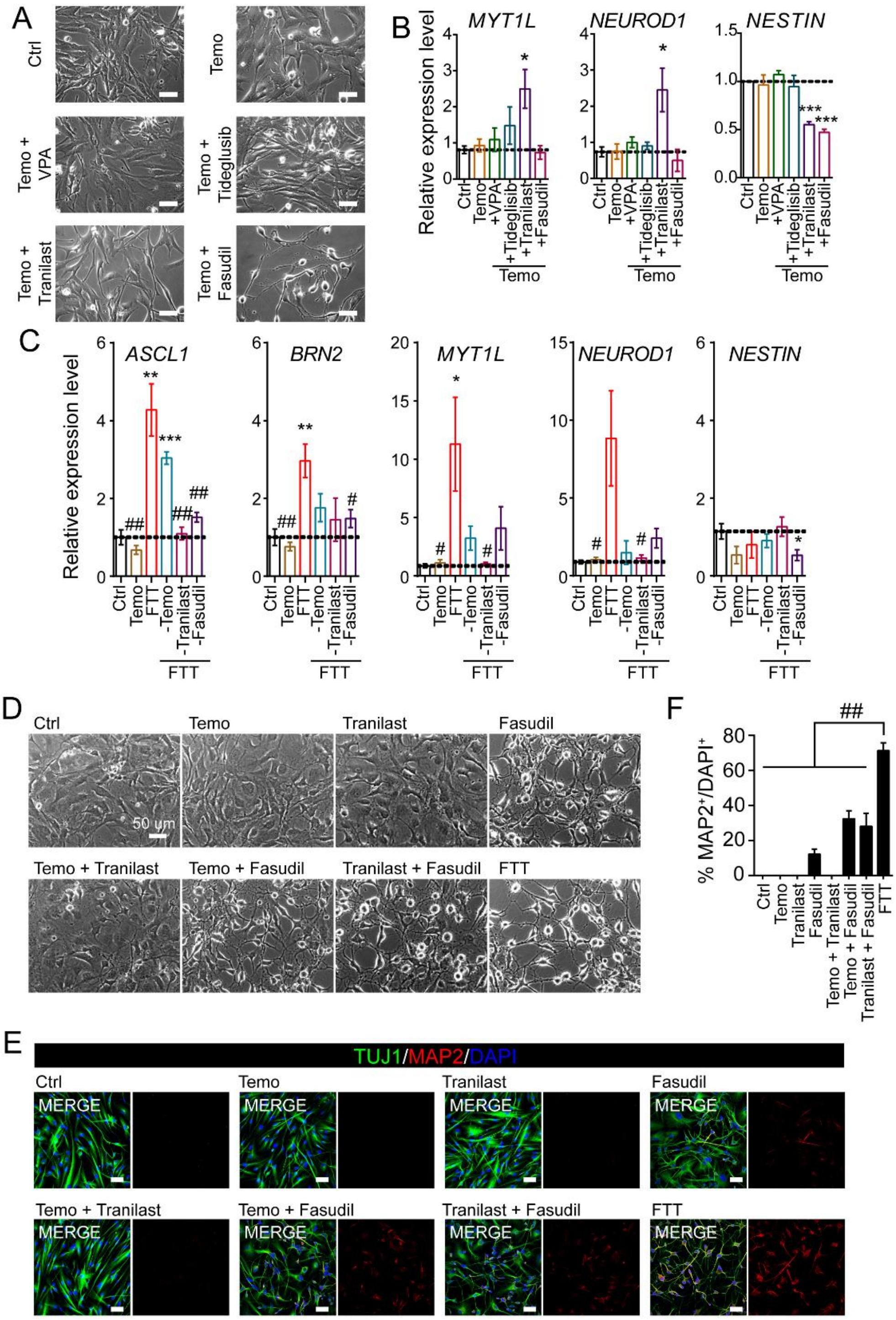

Figure S2. Identification of the neuronal reprogramming drug cocktail FTT.
(**A**-**B**) Screening for potential drug cocktail on serum cultured GBM cells. Cells treated with DMSO (Ctrl), Temo, or Temo plus another drug were analyzed on day 2. n=3 independent experiments. The *p* values versus control group were calculated by two-tailed student's t test. Serum cultured GBM-3 cells were used. The following final concentration was used: Temo, 50 μM; VPA, 0.5 mM; Tideglusib, 30 μM; Tranilast, 100 μM; and Fasudil, 50 μM.

(**C**) Effects of FTT cocktail on the expression of *ASCL1*, *BRN2*, *MYT1L*, *NEUROD1*, and *NESTIN*. Cells treated with DMSO (Ctrl), Temo, FTT, or any two drugs in FTT cocktail were analyzed on day 2. n=4 independent experiments. Serum cultured GBM-10 cells were used.
(**D**) Cell morphology of GBM cells with indicated treatment for 2 days. Serum cultured GBM-25 cells were used.
(**E**) Immunostaining of TUJ1 and MAP2 on GBM cells with indicated treatment for 6 days. Serum cultured GBM-25 cells were used.
(**F**) Quantification of reprogramming efficiency based on MAP2 expression and cell morphology in E.
Representative images of n=3 independent experiments are shown. Scale bar, 50 µm. Data are represented as mean ± SEM. Scale bar, 50 µm. * and # indicated statistical significance calculated with two-tailed student's t test versus control and FTT group respectively. *, p<0.05; **, p<0.01; ***, p<0.001; #, p<0.05; ##, p<0.01.

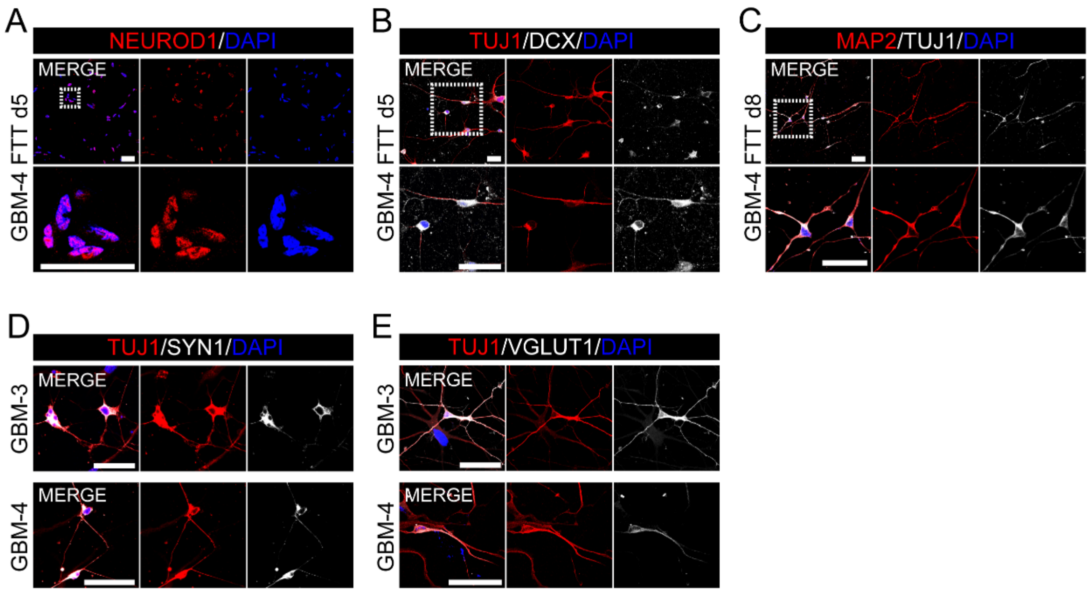

Figure S3. Reprogramming of serum cultured GBM cells into neuronal like cells by FTT.

(**A-C**) Immunostaining of NEUROD1 (A), TUJ1 (B, C), DCX (B), and MAP2 (C) on FTT treated GBM-4 cells on indicated days.

(**D-E**) Immunostaining of SYN1 (D) and VGLUT1 (E) on FTT-treated GBM cells on day 8.

Representative images of n=3 independent experiments are shown. Scale bar, 50 µm.

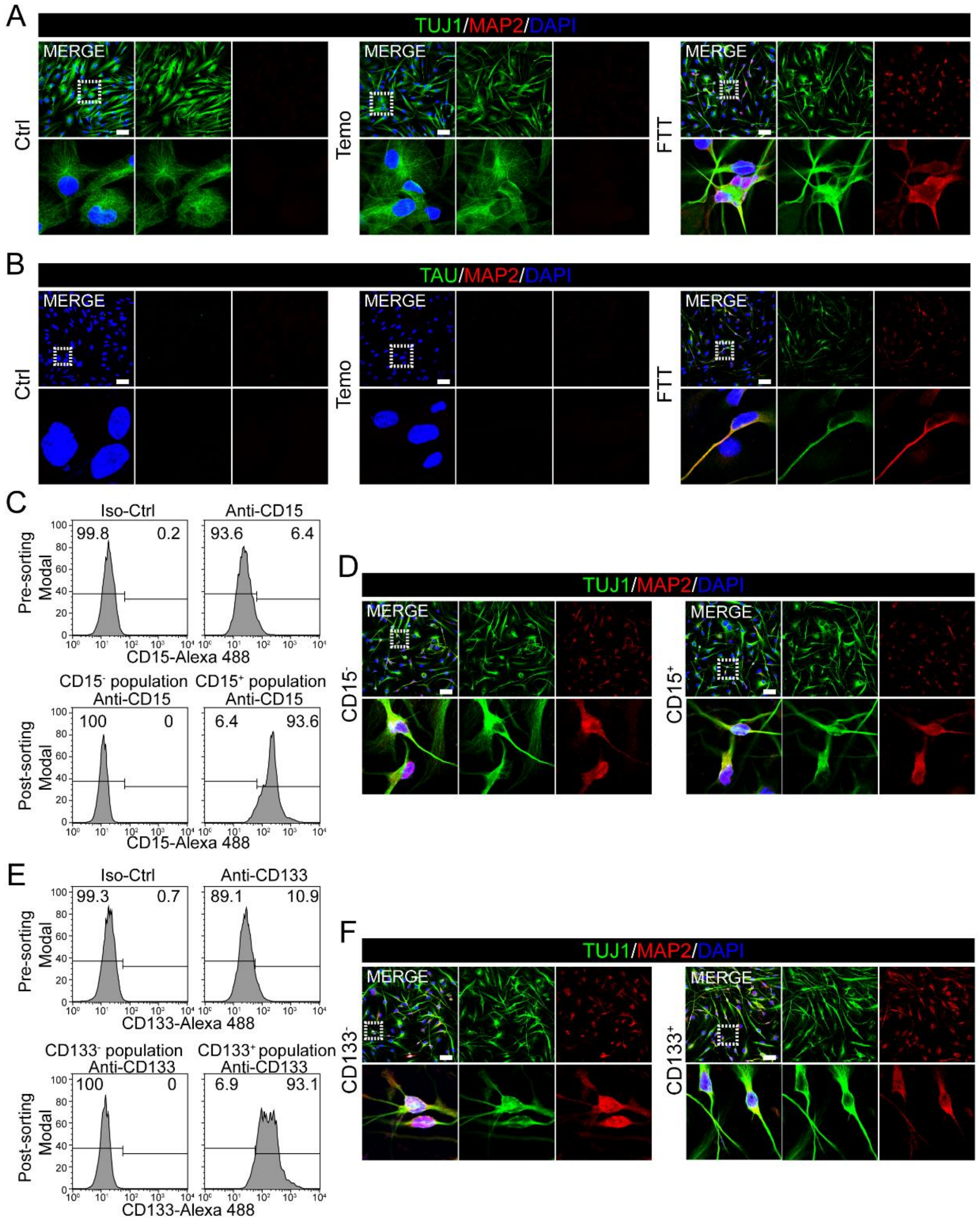

Figure S4. Induction of serum-free cultured TJ-17 cells into neuronal like cells by FTT.

(**A-B**) Immunostaining of TUJ1 (A), MAP2 (A, B), and TAU (B) on TJ-17 cells with indicated treatment for 6 days.

(**C**) Enrichment of CD15$^-$ and CD15$^+$ cells from serum-free cultured TJ-17 cells by FACS. The purity of post-sorted cells was analyzed by flow cytometry (lower panel).

(**D**) FACS-enriched CD15$^-$ and CD15$^+$ cells in C were treated with FTT and immunostained with TUJ1 and MAP2 on day 6.

(**E**) Enrichment of CD133$^-$ and CD133$^+$ cells from serum-free cultured TJ-17 cells by FACS. The purity of post-sorted cells was analyzed by flow cytometry (lower panel).

(**F**) FACS-enriched $CD133^-$ and $CD133^+$ cells in E were treated with FTT and immunostained with TUJ1 and MAP2 on day 6.
Representative results of n=3 independent experiments are shown. Scale bar, 50 μm.

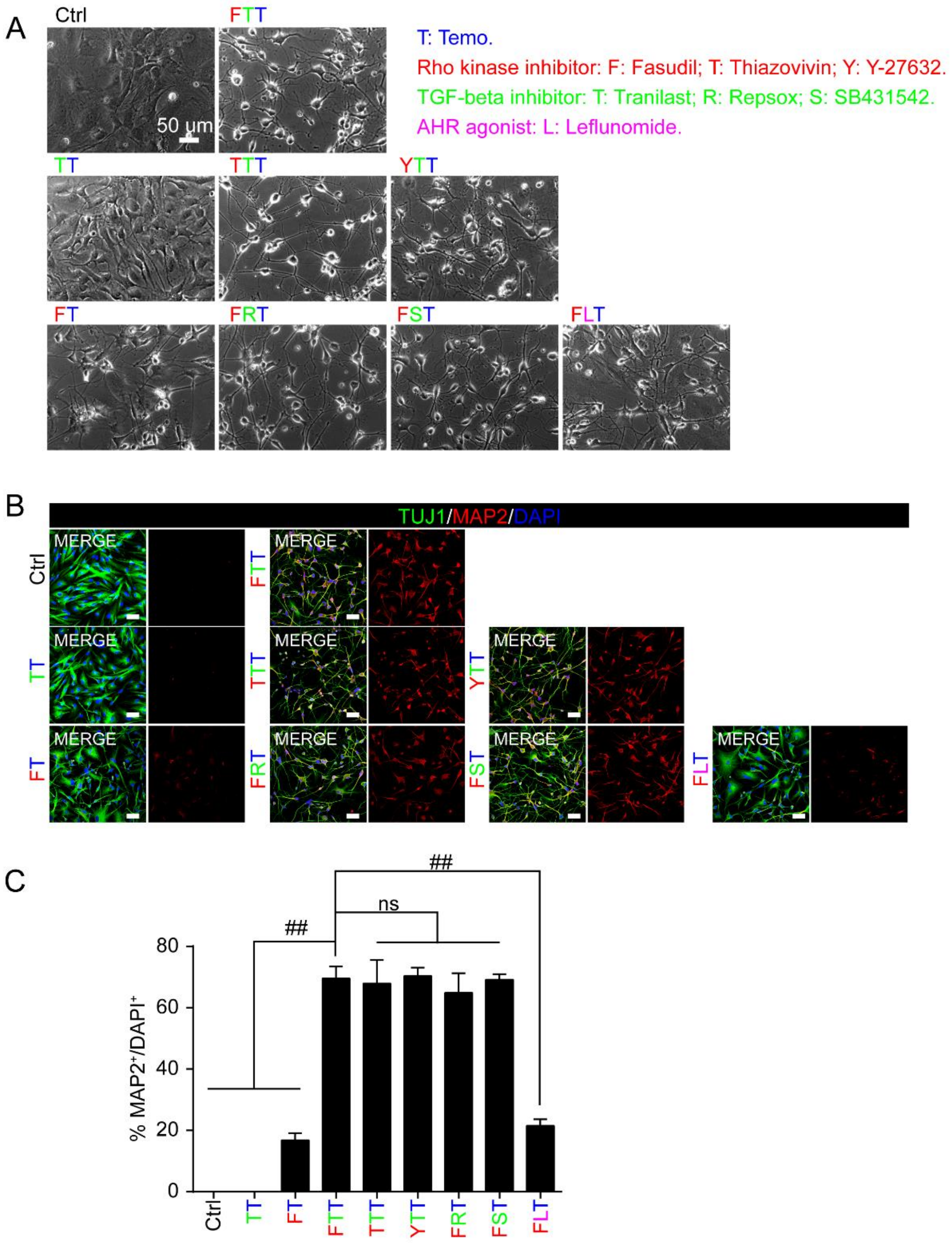

Figure S5. TGFβ and Rho kinase inhibition in neuronal reprogramming of GBM cells.

(**A**) Cell morphology of GBM cells with indicated treatment for 2 days. Thiazovivin and Y-23632 were used to replace Fasudil in FTT cocktail. Repsox, SB431542, and Leflunomide were used to replace Tranilast in FTT. GBM-25 cells were used.

(**B**) Immunostaining of TUJ1 and MAP2 on GBM cells with indicated treatment for 6 days. GBM-25 cells were used. Representative results of n=3 independent experiments are shown. Scale bar, 50 μm.

(**C**) Quantification of reprogramming efficiency based on MAP2 expression and cell morphology. # indicated statistical significance calculated with two-tailed student's t test versus FTT group. ##, $p<0.01$; ns, not significant.

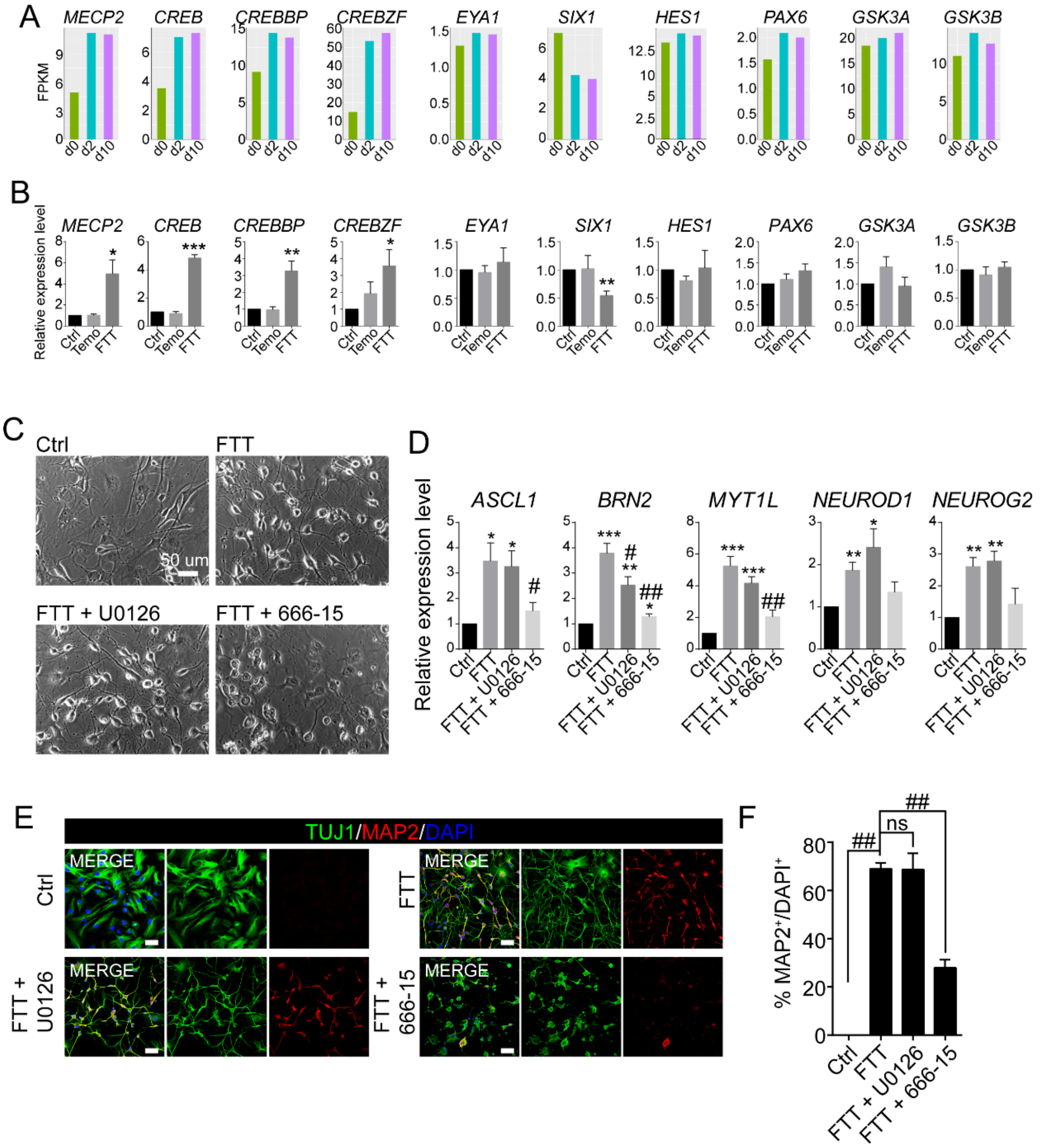

Figure S6. CREB signaling in FTT-mediated neuronal reprogramming.

(**A**) The FPKM values of indicated genes in GBM-17 cells (d0), and GBM-17 cells with FTT treatment for 2 days (d2) or 10 days (d10).

(**B**) RT-qPCR analysis of above-mentioned genes on GBM-17 cells with indicated treatment.

(**C**) Cell morphology of GBM-25 cells with indicated treatment for 2 days.

(**D**) RT-qPCR analysis of neuronal TFs on GBM-25 cells with indicated treatment.

(**E**) Immunostaining of TUJ1 and MAP2 on GBM-25 cells with indicated treatment for 6 days.

(**F**) Quantification of reprogramming efficiency based on MAP2 expression and cell morphology in E.

Scale bar, 50 µm. Data are represented as mean ± SEM. GBM-25 cells were used in C-F. * and # indicated statistical significance calculated with two-tailed student's t test

versus control and FTT group respectively. *, p<0.05; **, p<0.01; ***, p<0.001; #, p<0.05; ##, p<0.01; ns, not significant.

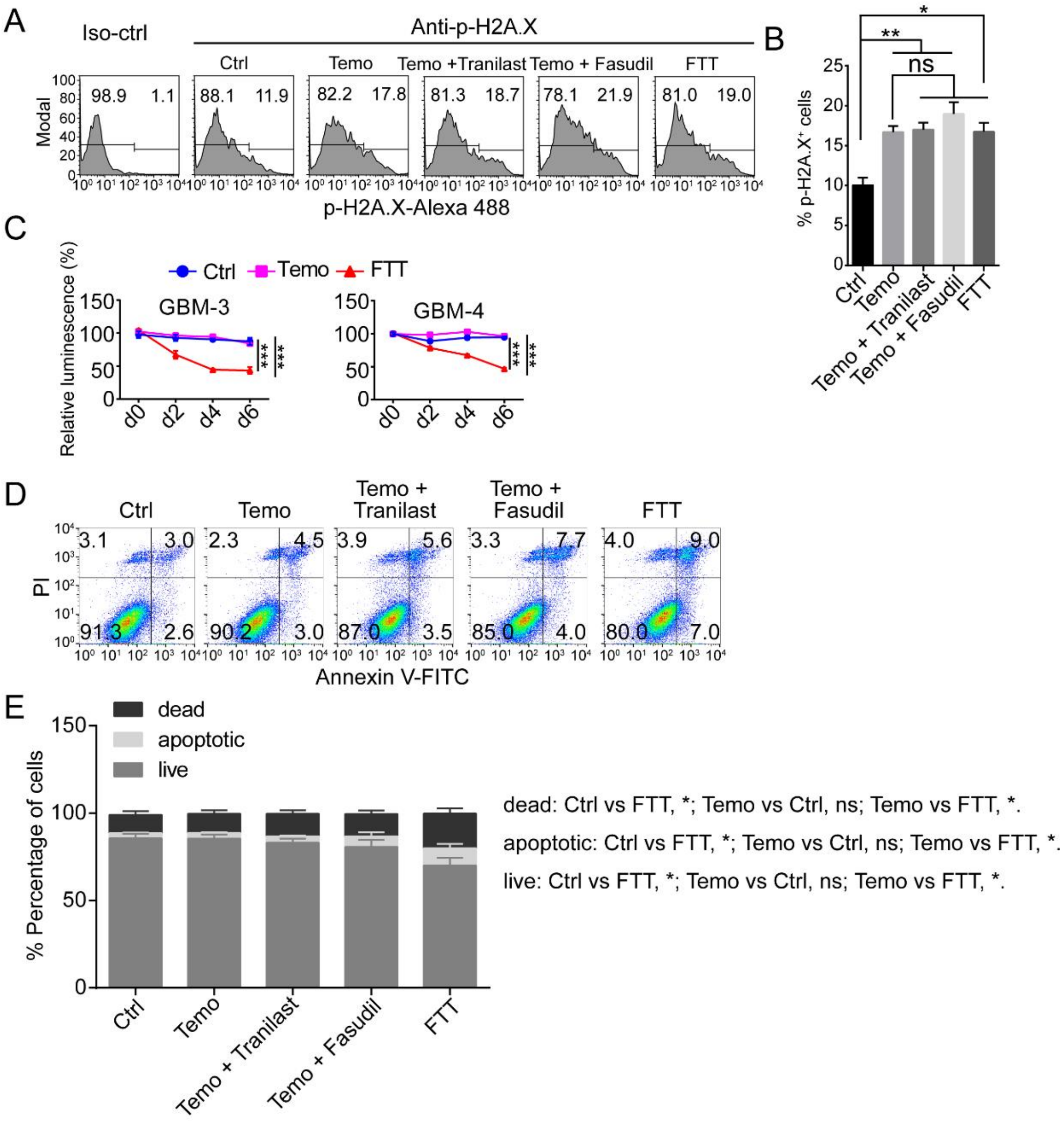

Figure S7. Further analysis of FTT on GBM cells.

(**A-B**) Flow cytometry analysis of p-H2A.X on GBM cells with indicated treatment for 24 hours. The quantitative results were shown in B. ns, not significant. GBM-17 cells were used.

(**C**) Cell viability measured by CellTiter-Glo on GBM cells with indicated treatment on indicated days. Two-way ANOVA and Tukey's multiple comparisons test were used to calculate statistical significances.

(**D-E**) Annexin-V and PI staining to measure cell death on GBM cells with indicated treatment for 24 hours. The quantitative results were shown in E. Live cells were Annexin-V$^-$PI$^-$ cells (lower left quadrant). Apoptotic cells were Annexin-V$^+$PI$^-$ cells (lower right quadrant). Dead cells were Annexin-V$^-$PI$^+$ and Annexin-V$^+$PI$^+$ cells (upper left and upper right quadrants). The experiments were performed on GBM-17 cells.

Data are represented as mean ± SEM. n=3 independent experiments. *, p<0.05; **, p<0.01.

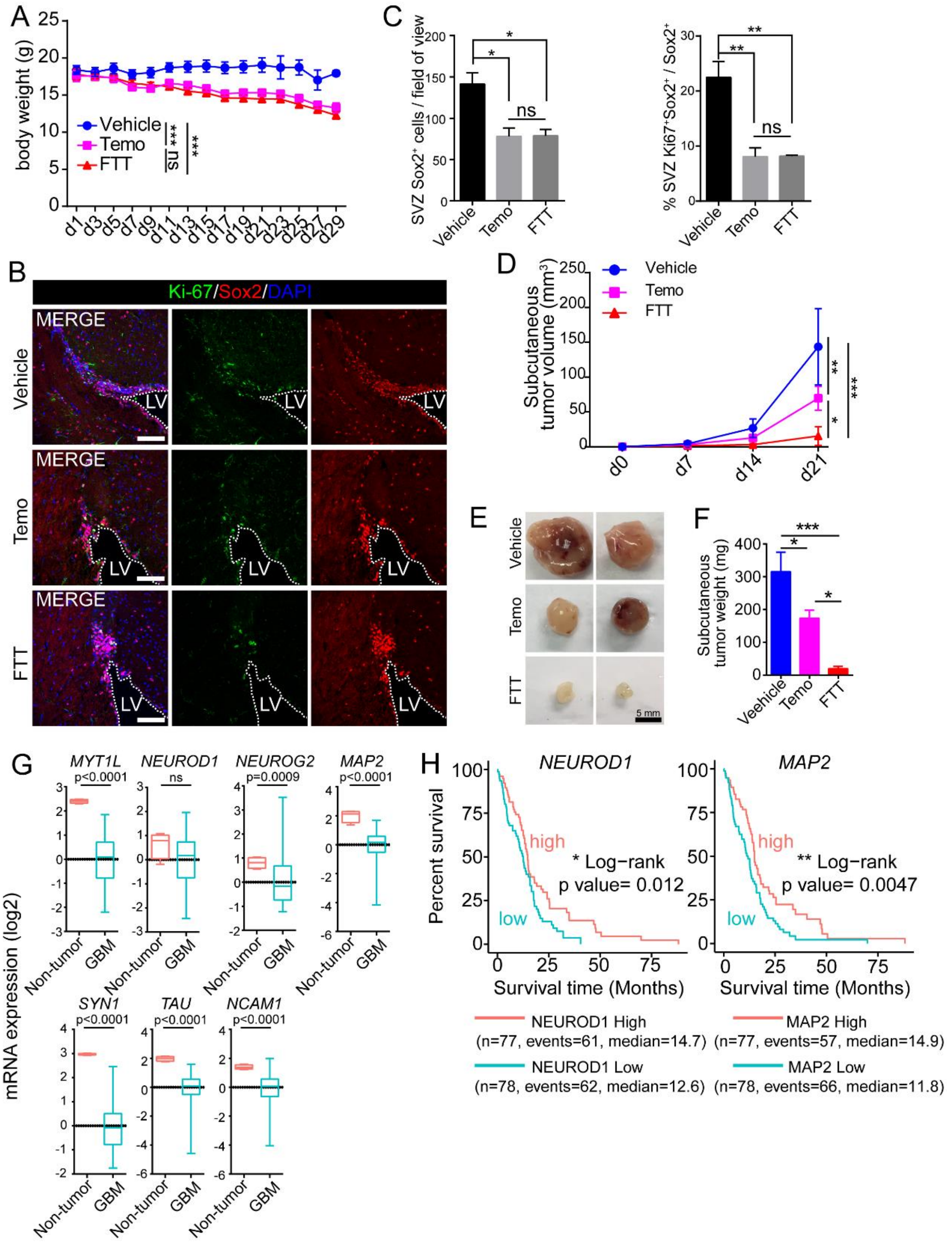

Figure S8. The effects of FTT cocktail in vivo and expression of neuronal genes in GBM patients.

(**A**) Body weight of mice with indicated treatment. Two-way ANOVA and Tukey's multiple comparisons test were used to calculate statistical significances.

(B-C) Immunostaining of Sox2 and Ki67 on SVZ NPCs in mice with indicated treatment. The number of SVZ $Sox2^+$ cells per field of view and the percentage of $Ki67^+$ in SVZ $Sox2^+$ cells are quantified in C. LV, lateral ventricle. Scale bar, 50 μm.

(**D**-**F**) Subcutaneous tumor volume and weight in mice with indicated treatment. n=5 mice per group. Data in E and F were derived on day 27, when mice were sacrificed. Scale bar, 5 mm.

(**G**) Differences in overall survival for GBM patients with differential expression of *NEUROD1* or *MAP2*. GBM of all subtype were included and median cutoff was applied.

(**H**) Box and Whisker chart showing the differential expression of neuronal genes (*MYT1L*, *NEUROD1*, *NEUROG2*, *MAP2*, *SYN1*, *TAU*, and *NCAM1*) in non-tumor and GBM tissues. Two-tailed unpaired t test with Welch's correction was used for *MYT1L*, *NEUROG2*, *SYN1*, *TAU*, and *NCAM1*, as the variances among non-tumor and GBM groups were significantly different for these genes (F test, *MYT1L*, p=0.0016; *NEUROG2*, p=0.0334; *SYN1*, p=0.0002; *TAU*, p= 0.0127; and *NCAM1*, p=0.0099). Two-tailed unpaired t test was used for *NEUROD1* and *MAP2*, as the variances among non-tumor and GBM groups were not significantly different for these two (F test, *NEUROD1*, p=0.3625; *MAP2*, p=0.2065). n= 4 and 156 samples for non-tumor and GBM. Data in G and H were accessed via GlioVis (gliovis.bioinfo.cnio.es).

Data are represented as mean ± SEM. *, $p<0.05$; **, $p<0.01$; ***, $p<0.001$; ns, not significant.

Supplementary Tables
Table S1. Specimen used in this study

GBM-3, GBM-4, GBM-10, GBM-11, GBM-17, and GBM-25 cells were isolated from patient samples in this study. The pathological information for these samples were listed below.

GBM cells	Age	Pathological diagnosis	Pathological examination results	
			**Positive**	**Negative**
GBM-3	61	GBM (WHO, grade IV), deep right temporal lobe	GFAP, OLIG2, ATRX, MIB-1 (20%+), NESTIN	P53, IDH-1, NEUN, H3K27M
GBM-4	62	GBM (WHO, grade IV), left parietal lobe	GFAP, OLIG2 (-/+), ATRX, MIB-1 (10%+)	P53, IDH-1, NEUN, H3K27M
GBM-10	54	GBM (WHO, grade IV), right frontal lobe	GFAP, OLIG2, ATRX, NEUN, Ki67 (30%+), SYN (+/-), NF (-/+)	IDH-1, EMA, CD34, INA
GBM-11	51	GBM (WHO, grade IV), right frontal lobe, right temporal lobe	GFAP, OLIG2, ATRX, MIB-1 (35%+)	P53, IDH-1, INA, NEUN, H3K27M
GBM-17	32	GBM (WHO, grade IV), right frontal lobe	GFAP, ATRX, MIB-1 (20%+)	OLIG2, P53, IDH-1, DES, NEUN, H3K27M, CK
GBM-25	39	GBM (WHO, grade IV), right temporal lobe	OLIG2, P53, Ki67(12%), H3K27M(+/-), H3K37me3(-/+)	IDH-1, GFAP, ATRX, INA, NEUN

Table S2. RT-qPCR primers used in this study.

Gene	Forward	Reverse
ASCL1	CAAGAGAGCGCAGCCTTAG	GCAAAAGTCAGTGCTGAACG
BRN2	AATAAGGCAAAAGGAAAGCAACT	CAAAACACATCATTACACCTGCT
MYT1L	CAATGGAAAGGGATTTTAAGCA	TTTGAGATTATGTACCAACGTTAGATG
NEUROD1	GTTATTGTGTTGCCTTAGCACTTC	AGTGAAATGAATTGCTCAAATTGT
NEUROG2	TCAGACATGGACTATTGGCAG	GGGACAGGAAAGGGAACC
NESTIN	CAACAGCGACGGAGGTCTC	GCCTCTACGCTCTCTTCTTTGA
CCNE1	GCCAGCCTTGGGACAATAATG	CTTGCACGTTGAGTTTGGGT
CDK2	CCAGGAGTTACTTCTATGCCTGA	TTCATCCAGGGGAGGTACAAC
CDK4	ATGGCTACCTCTCGATATGAGC	CATTGGGGACTCTCACACTCT
CDK6	CCAGATGGCTCTAACCTCAGT	AACTTCCACGAAAAAGAGGCTT
CDC25C	TCAAGTCTTCGCCTGTGTCC	CTGAGCCAGAGCTTCCTTCC
TPX2	ATGGAACTGGAGGGCTTTTTC	TGTTGTCAACTGGTTTCAAAGGT
RRM2	CACGGAGCCGAAAACTAAAGC	TCTGCCTTCTTATACATCTGCCA
PXDN	AATCAGAGAGATCCAACCTGGG	AATGCTCCACTAGGTATCCTCTT
MELK	TCTCCCAGTAGCATTCTGCTT	TGATCCAGGGATGGTTCAATAGA
TNC	TCCCAGTGTTCGGTGGATCT	TTGATGCGATGTGTGAAGACA
MECP2	TGACCGGGGACCCATGTAT	CTCCACTTTAGAGCGAAAGGC
CREB	ATTCACAGGAGTCAGTGGATAGT	CACCGTTACAGTGGTGATGG
CERBBP	CAACCCCAAAAGAGCCAAACT	CCTCGTAGAAGCTCCGACAGT
CREBZF	ATGAGGCATAGCCTGACCAAG	GCAGCGAACAAGTTGCAGC
EYA1	CACCACAGATTTACCCTTCCAAC	GTACGTGGCATAGGCTGTAGC
SIX1	CTGCCGTCGTTTGGCTTTAC	GCTCTCGTTCTTGTGCAGGT
HES1	TCAACACGACACCGGATAAAC	GCCGCGAGCTATCTTTCTTCA
PAX6	TGGGCAGGTATTACGAGACTG	ACTCCCGCTTATACTGGGCTA
GSK3A	GGAAAGGCATCTGTCGGGG	GAGTGGCTACGACTGTGGTC
GSK3B	GGCAGCATGAAAGTTAGCAGA	GGCGACCAGTTCTCCTGAATC
HPRT	CCTGGCGTCGTGATTAGTGAT	AGACGTTCAGTCCTGTCCATAA
